# COSMOS: a platform for real-time morphology-based, label-free cell sorting using deep learning

**DOI:** 10.1038/s42003-023-05325-9

**Published:** 2023-09-22

**Authors:** Mahyar Salek, Nianzhen Li, Hou-Pu Chou, Kiran Saini, Andreja Jovic, Kevin B. Jacobs, Chassidy Johnson, Vivian Lu, Esther J. Lee, Christina Chang, Phuc Nguyen, Jeanette Mei, Krishna P. Pant, Amy Y. Wong-Thai, Quillan F. Smith, Stephanie Huang, Ryan Chow, Janifer Cruz, Jeff Walker, Bryan Chan, Thomas J. Musci, Euan A. Ashley, Maddison (Mahdokht) Masaeli

**Affiliations:** 1 Deepcell Inc; 4025 Bohannon Dr., Menlo Park, CA 94025 USA; 2 Department of Medicine, Genetics, & Biomedical Data Science, Stanford University, Stanford, CA USA

**Keywords:** Biotechnology, Machine learning, Cellular imaging

## Abstract

Cells are the singular building blocks of life, and a comprehensive understanding of morphology, among other properties, is crucial to the assessment of underlying heterogeneity. We developed Computational Sorting and Mapping of Single Cells (COSMOS), a platform based on Artificial Intelligence (AI) and microfluidics to characterize and sort single cells based on real-time deep learning interpretation of high-resolution brightfield images. Supervised deep learning models were applied to characterize and sort cell lines and dissociated primary tissue based on high-dimensional embedding vectors of morphology without the need for biomarker labels and stains/dyes. We demonstrate COSMOS capabilities with multiple human cell lines and tissue samples. These early results suggest that our neural networks embedding space can capture and recapitulate deep visual characteristics and can be used to efficiently purify unlabeled viable cells with desired morphological traits. Our approach resolves a technical gap in the ability to perform real-time deep learning assessment and sorting of cells based on high-resolution brightfield images.

## Abstract (web summary)

Computational Sorting and Mapping of Single Cells (COSMOS) is a cloud-enabled platform that performs real-time cell imaging, analysis, and sorting using deep learning-based morphology representations.

## Introduction

Technological advances have made single-cell characterization at the genomic, transcriptomic, and proteomic levels a reality, yielding comprehensive cell atlases with detailed molecular data on hundreds of cell types from multiple organisms^1–5^. Yet, tools for assessing high-dimensional cell morphology at single-cell resolution have not kept pace with advancements in molecular characterization. Cell morphology has been used by pathologists and clinicians for years as the gold standard for disease diagnosis and prognosis^6^. A growing body of evidence shows morphology serves as a readout of genomic and/or functional states, including gene expression, metastatic potential, and mechanism(s) of drug response^7,8^. Beyond epitope and biomarker-based cell capture, fluorescence-activated cell sorting (FACS) data provides useful morphology-related information, including cell size and granularity^9,10^, further highlighting the value of morphology quantification. To complement current technologies that assess cell biology at single-cell resolution, the ability to directly quantify morphology using multiple dimensions and sort populations of interest for downstream analysis is critical.

Recent efforts in image-based cell sorting, which deploy high-speed image capture of cells in flow, have recognized the potential to discern and study visual information of cells^10–17^. However, these innovations are limited by dependence on biomarker staining^14^ or deformability assays^12^, which compromise cell viability for downstream functional assays. Some approaches that use feature engineering of low-resolution or reconstructed images may not capture the entirety of complex morphological information. Accordingly, the number of morphological traits that are simultaneously characterized may be limited^13,16^. Some techniques require manual processes to define a small number of features to quantify and sort cells based on morphology. For instance, sorting decisions are made based on population gating on a small number of specific spectral signals that allude to the morphological characteristics of cells^14^. In a previous report, image features based on fluorescent signals are used to establish a hierarchical gating strategy for cell sorting that is fine-tuned to a specific application^14^. The application of deep learning and machine intelligence can simultaneously deepen and generalize the characterization of image traits. Machine intelligence has allowed accurate classification of cells on pathology slide images^18^, including recapitulation of immunohistochemistry signals from light microscopy alone^19^. One group combined shallow convolutional neural network (CNN) classification with a sorting device to classify and isolate a limited set of cells with clear morphological differences based on reconstructed cell images^13,20^. Despite tremendous progress and insights from recent work, real-time deep learning classification and sorting decisions based on high-resolution images have remained prohibitively challenging. Machine learning approaches for single-cell sorting remain limited to small datasets due to technical constraints in integrating deep neural network architectures with real-time cell sorting.

**Fig. 1:**
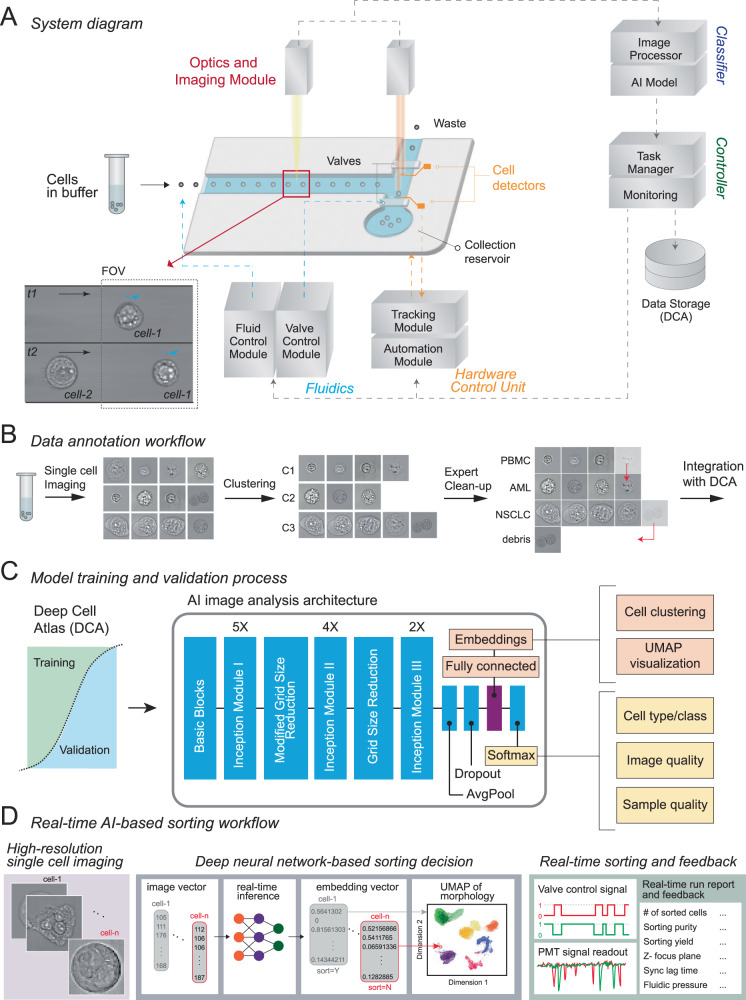
COSMOS platform workflow and schematic. **a** System diagram. The hardware includes Fluidics (Fluid Control and Valve Control Modules), Optics and Imaging Module, and Hardware Control Unit for auto-focusing and -alignment (Tracking and Automation Modules). The software includes Classifier, Controller, and Data Storage modules. **b** Data annotation workflow. AI-assisted image annotation software is used to cluster individual cell images. A human expert uses the labeling tool to adjust and batch-label the cell clusters. In the example shown, one acute myeloid leukemia (AML) cell was misclustered with a group of PBMCs, and an image showing debris was misclustered with a group of NSCLC cells. These errors are corrected by the “Expert Clean-up” step. The annotated cells are then integrated into the Deep Cell Atlas (DCA). **c** Model training and validation process. Annotated cell images are split into independent training and validation image sets. AI image analysis depicting the InceptionV3 model architecture is shown. The fully connected layer of the architecture is used for cell clustering and UMAP visualization. The softmax layer generates per-cell classification and prediction probabilities. **d** Real-time AI-based sorting workflow. Images of single cells are converted to a vector, and a user-selected classifier assesses each cell. The embedding vector generated by the model is used to visualize the sample profile (e.g., UMAP depiction is drawn based on the embeddings). Real-time inferences guide a sorting decision based on user preferences. See “Methods” for comprehensive details.

We present Computational Sorting and Mapping of Single Cells (COSMOS), a cloud-enabled platform that performs real-time cell imaging, analysis, and sorting using deep learning-based morphology representations (Fig. 1, Supplementary Figs. 1 and 2). The platform classifies and sorts live cells based on high-resolution brightfield images, thereby enabling quantification and mapping of cell morphology as a biological descriptor^11^. COSMOS is outfitted with a data infrastructure to host a large atlas of annotated single-cell images, a library of deep learning models with the computational capacity to classify label-free brightfield images, and fluidics hardware to isolate target cells based on model classifications (Fig. 1). Several approaches that apply deep learning to cell sorting rely on readouts that can be reconstructed into an image, rather than actual images of cells. A potential drawback of this approach is lower information content and resolution. Here, we analyzed and sorted cells based on high-resolution brightfield images of single cells using deep learning. This approach is critical in multiple ways. First, a model that can capture minute variations in images at this resolution is demanding to train and requires relatively large datasets. For instance, many studies developed a model using thousands of images^12,20,21^, compared to millions of images in our study. The selected model architecture (InceptionV3) is deep enough to represent high-resolution images of multiple complex objects. Second, running inferences on this deep architecture in real-time to sort cells using high-resolution image data is computationally challenging. To achieve real-time classification, computation is distributed across an ultra-high-speed camera, microcontroller, CPUs, and GPUs with an optimized version of the Inception architecture. Third, we designed the microfluidics system to keep cells in an ultra-tight focus range. This is critical to the performance of a system that captures high-resolution images and is sensitive to slight variations of focus. Finally, we used dimensionality reduction techniques and high-dimensional data visualization analysis tools (e.g., UMAP) to enable intuitive interpretation of deep morphological representations^22^. Detailed descriptions of instrument specifications, including latency time, sorting purity, cell flow rate, and base code, can be found in Supplementary information.

Using multiple cell lines and solid tumor biopsy samples, we show that the COSMOS tool can be used to (1) visualize deep morphological distinctions across biological samples, (2) discriminate and enrich specific cells of interest with high accuracy in a label-free manner, and (3) provide a link between morphology and molecular biology through delivering minimally perturbed cells with distinct morphological characteristics. This platform presents a framework with promising applications in the discovery of inherent morphological phenotypes and the ultimate integration of morphology with multi-omics data analysis.

## Results

### Cell clusters in the embedding space

**Fig. 2:**
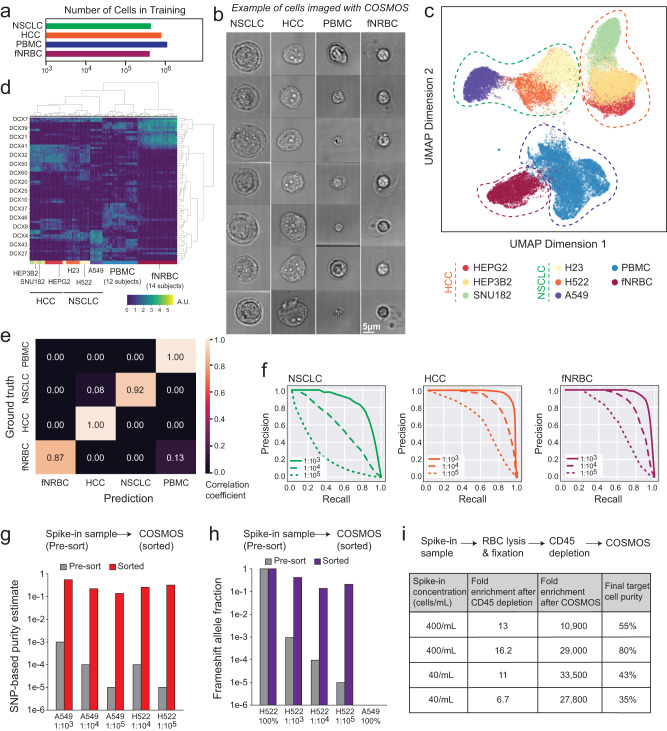
Quantitative morphological assessment of single cells and performance of Circulating Cell Classifier in identifying cells. **a** Number of cell images used in the training set for each of the cell classes. **b** Representative images of NSCLC, HCC, PBMC, and fnRBC cells captured by COSMOS. **c** UMAP projection of cell embeddings sampled from cell classes analyzed by the model. Each point represents a single cell. **d** Heatmap representation of the embedding space. Each column is a single cell for HCC, NSCLC, PBMC, and fNRBC classes. Each row is an embedding dimension. A.U. arbitrary units. **e** Confusion matrix representing Circulating Cell Classifier prediction accuracy (*x*-axis) versus ground truth (*y*-axis) on the validation set. **f** Estimated precision-recall curves at different proportions for positive selection of NSCLCs, HCCs, and fNRBCs in PBMC background. Precision: estimated purity and recall to the yield of target cells based on an in silico mixture of datasets of known cell types. Three curves are shown for different target cell proportions: 1:1000, 1:10,000, and 1:100,000. **g** Purity of pre-sorted and sorted cells estimated by comparing allele fractions with an SNP panel to the known genotypes at indicated spike-in ratios. **h** Frameshift mutation assay in the *TP53* gene (c.572_572delC). **i** Indicated number of A549 cells were spiked into whole blood, and samples were processed on COSMOS for malignant cell identification and sorting. Sorted cell purity and fold enrichment quantified by SNP analysis.

To demonstrate COSMOS’s ability to identify different cell types based on morphology, we trained a CNN model using peripheral blood mononuclear cells (PBMCs), fetal nucleated red blood cells (fnRBC), non-small cell lung cancer (NSCLC), and hepatocellular carcinoma (HCC) cell lines using the indicated number of cells–termed the “Circulating Cell Classifier” (Fig. 2a). Representative images of each of these four classes are shown in Fig. 2b, Supplementary Fig. 3a. Low-dimensional representations that capture AI descriptors of cell images, “embeddings”, were extracted and plotted as Uniform Manifold Approximation and Projection (UMAPs). Distinct cell types (e.g., NSCLC vs HCC) clustered separately from one another (Fig. 2c). Within NSCLC and HCC clusters, the three cell lines were clustered separately, suggesting morphological differences not only between cell types but also between cell lines within the same cell type (e.g., H23, H522, and A549 for NSCLC). PBMC samples showed high morphological variation, consistent with the heterogeneous composition of PBMCs (e.g., lymphocytes, monocytes, and dendritic cells). HEP3B2 and H23 cell lines are equivalent in size but plot separately in embedding space, indicating that additional AI vectors beyond cell size contribute to the morphological differences driving this separation (Fig. 2d, Supplementary Fig. 3b, c).

Next, we assessed COSMOS’s ability to distinguish healthy cells in varying cell states using T cell activation and differentiation as examples. Embedding projections shown using t-distributed stochastic neighbor embedding (tSNE) plots demonstrate distinct clusters between activated and naive T cells (Supplementary Fig. 4a). Additionally, a time-course (Day 0–5) study of T cell differentiation shows morphological properties shift with changing cell state and identity, as reflected in projected embeddings (Supplementary Fig. 4b). These results suggest T cell activation and differentiation state, in addition to PBMCs versus malignant cells, can be distinguished by deep morphological analysis of brightfield images (Supplementary Fig. 4).

### In silico evaluation of model performance and cell enrichment

In silico analysis was performed to examine the accuracy of the Circulating Cell Classifier in classifying the four cell types above in a supervised fashion. The confusion matrix shows that model prediction for fNRBCs, HCCs, NSCLCs, and PBMCs matches the actual cell class at 87%, 100%, 92%, and 100%, respectively (Fig. 2e).

We tested COSMOS’s ability to identify low-abundance NSCLCs, HCCs, and fnRBCs from a PBMCs background using positive or negative selection from an in silico mixture of known cell type datasets in varying proportions. For the NSCLC cell class, classifier performance yielded an area under curve (AUC) of 0.9842 for positive selection and 0.9996 for negative selection. For the HCC cell class, AUC was 0.9986 and 0.9999 for positive and negative selection, respectively (Supplementary Fig. 5a, b). While we demonstrated low false positive rates (FPR) for both modes of classification, positive selection in both cases enabled higher yields at low FPR < 0.0004. For the fnRBC cell class, we assessed only the mode of positive selection, which yielded an AUC of 0.97 (Supplementary Fig. 5c). This in silico analysis shows that even at 1:100,000 dilution, the model supports the detection of target cells at >70% precision (positive predictive value or post-enrichment purity) and 50% recall (sensitivity) in both the fnRBC and HCC samples, while recall drops to 15% for NSCLC class (Fig. 2f, Supplementary Fig. 6).

### Performance of target cell enrichment

Cells classified as target cells can be sorted into a collection reservoir for validation and/or further downstream analysis (see “Methods”). Cell sorting is performed using a pair of pneumatic microvalves controlled by a digital signal processing (DSP)-based microcontroller (see “Methods”). The valve timing can be adjusted based on desired purity or yield, depending on cell rate. Supplementary Fig. 1 shows the tradeoff between purity and yield based on cell rate, in addition to valve timing adjustments required for desired purity and yield. For example, at 3000 cells/min sorting rate, at ~80% yield, ~60% purity can be achieved with the valve window adjusted to ~15 ms (Supplementary Fig. 1). To biologically validate our in silico analysis, we performed simultaneous classification and enrichment experiments by spiking NSCLC cell lines and fnRBCs from 1:1000 to 1:100,000 into PBMCs and estimated sorted cell purity using single nucleotide polymorphism (SNP) assays. A549 and H522 cells exhibited similar enrichment and purity (Fig. 2g, Supplementary Fig. 7, Supplementary Table 1), even though the classifier was trained on A549 cells. At a 1:100,000 spike-in ratio, 20% and 30–33% purities corresponding to 13,904 and 30,000–32,500-fold enrichment were obtained for A549 and H522 cells, respectively.

We next assayed for a frameshift mutation in *TP53* (c.572_572delC), which is homozygous in H522 and wildtype in A549^23^ (Fig. 2h, Supplementary Table 2). Even at 1:100,000 spike-in ratio, the mutation was present at 23% allele fraction in DNA extracted from enriched cells, suggesting functionally important mutations are detected even when cells containing them are at <1:100,000 concentrations.

To further test the performance of identifying and isolating low-abundance cells, A549 cells were spiked into whole blood at 40 cells/mL and 400 cells/mL. To simulate negative enrichment workflows by depleting cells other than cells of interest, RBC lysis and CD45+ cell depletion were performed prior to COSMOS processing. SNP analysis of sorted samples had final purities (and fold enrichment) of 55% (>10,900x) and 80% (>29,000x) for 400 cells/mL replicates and 43% (>33,500x) and 35% (>27,800x) for 40 cells/mL replicates (Fig. 2i, Supplementary Table 3, Supplementary Fig. 8).

### Gentle and label-free sorting yields viable cells with unaltered scRNA-Seq profiles

We tested whether live cells sorted with COSMOS maintain viability and are amenable to downstream scRNA-Seq analysis with minimal changes to the transcriptomic profile. We found COSMOS had minimal or no impact on cell viability among cell lines and primary cells tested (Supplementary Table 4). Single-cell gene expression profiles between unprocessed and COSMOS-processed PBMCs by scRNA-Seq showed a high correlation between gene expression profiles using both a targeted immune response panel (R^2^ = 0.97) and whole transcriptome amplification (WTA) (R^2^ = 0.983), indicating that COSMOS processed cells are transcriptionally comparable to unprocessed cells (Supplementary Fig. 9a–c). We next examined the impact of COSMOS on gene expression profiles using a cell type known to be sensitive to cell processing^24^. We used FACS, a common lab technique involving cell processing, as a reference point for gene expression alterations. (Supplementary Fig. 9d). Comparison of gene expression profiles of viable cells that were COSMOS-sorted cells showed fewer up- or down-regulated genes relative to control cells compared to FACS (Supplementary Fig. 9e, Supplementary Table 5). Additionally, COSMOS processing resulted in less activation in genes involved in multiple immune cell activation pathways and neutrophil degranulation pathways (Supplementary Fig. 9f).

### Application of COSMOS on identification and enrichment of NSCLC tumor cells from dissociated solid tissue biopsies

**Fig. 3:**
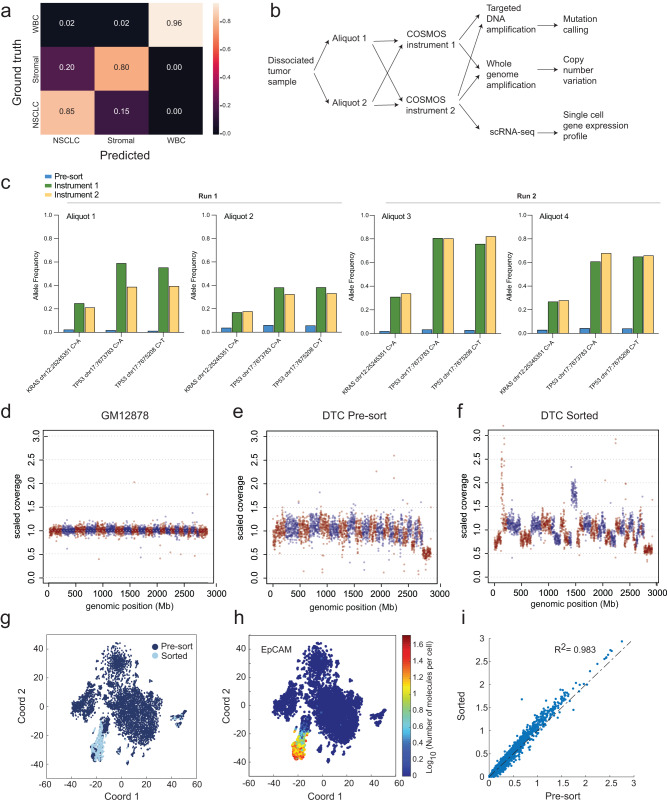
Performance of COSMOS in identifying and isolating target cells. **a** Confusion matrix representing Lung Cancer Classifier prediction accuracy (*x*-axis) vs the ground truth (*y*-axis) on the validation dataset. **b** Workflow schematic of COSMOS sorting and downstream molecular analysis of DTCs applied to (**c**–**i**). **c** Allele frequency of *KRAS* mutation (Chr12:25245351C>A) and *TP53* mutations (chr17:7673783C>A and chr17:7675208C>T) detected in four pre-sorted and sorted DTC aliquots following processing on two COSMOS instruments across two experimental runs. **d**–**f** WGA and CNV analysis of pre-sorted and sorted samples. Each data point represents 1 Mb bin. Red and blue colors indicate different chromosomes. GM12878 genomic DNA was used as a baseline control for copy number normalization. **g** scRNA-Seq gene expression t-SNE plot with all 924 feature-selected genes for pre-sorted (dark blue) and post-sorted (light blue) shown as an overlay. **h** Pseudo-color EpCAM gene expression level. **i** Gene expression correlation plot of mean (log_10_(molecules per cell per gene)) for pre-sorted and sorted cells from the EpCAM^+^/PTPRC(CD45)^−^ cluster. Each data point is a gene. The gene expression correlation coefficient (R^2^) was 0.98.

To apply COSMOS to a real-world use case of identifying and enriching NSCLC tumor cells from primary dissociated tumor cell (DTC) tissue, we trained a separate model termed Lung Tumor Classifier (Supplementary Table 6). The Lung Tumor Classifier confusion matrix shows the model prediction for NSCLCs, stromal cells, and white blood cells (WBC) matches the ground truth cell class at 82%, 78%, and 96%, respectively (Fig. 3a, Supplementary Fig. 10a).

We validated model accuracy in identifying malignant cells using three NSCLC DTC samples, which showed high concordance to the percent malignant cells determined by scRNA-Seq for low (2.2% vs 4.6%), medium (12% vs 16.8%), and high (40% vs 46.7%) malignant cell purities (Supplementary Fig. 10b–g). As confirmation that COSMOS can enrich malignant cells from tumor tissue, we sorted cancer cells from a DTC sample of a stage IIB NSCLC patient. The sample was split into two aliquots, which were run on separate instruments. Sorted cells were split into multiple fractions for molecular analysis (Fig. 3b, Supplementary Table 7). Using a targeted lung cancer panel, we found one *KRAS* and two *TP53* mutations, with sorted sample allele frequencies increased from <3% up to ~20% and 1–6% up to ~80%, respectively (Fig. 3c). Accordingly, mutations initially at low levels were significantly enriched, suggesting COSMOS enrichment captured mutational heterogeneity of the pre-sorted sample and improved confidence in mutation calling beyond any technical noises for low tumor content samples. Additionally, COSMOS yielded a significant increase in sensitivity of copy number variation (CNV), in which chr8q was amplified (location of *MYC* and *PRDM14*^25^) (Fig. 3d–f).

We next confirmed the identities of the sorted cells and compared the scRNA profiles to pre-sorted cells using a WTA workflow (Fig. 3b). In the DTC sample, 6.71% pre-sorted and 94.16% sorted populations were EpCAM+/CD45−, indicating high purity in the sorting capability (Fig. 3g, h, Supplementary Fig. 11a, b)^26,27^. Additional NSCLC transcriptional biomarkers (*KRT18, CEACAM6, HOPX, FOXC1, CDH1*) were also enriched in sorted samples (Supplementary Fig. 11a, b)^27–29^. Sorted and pre-sorted cells from the EpCAM+/CD45− cluster showed strong gene expression correlation (R^2^ = 0.98), and overlapped in all subclusters, suggesting sorting was unbiased for the EpCAM+ population and did not change gene expression profile due to the gentle microfluidic flow (Fig. 3i and Supplementary Fig. 11c, d). A close examination of 166 stress and apoptosis-related genes (a preloaded gene set from BD^TM^ Data View v1.2.2 software (BD Biosciences, CA)) also did not show differences in the sorted cells compared to the pre-sorted sample (Supplementary Fig. 11e–g). Together, this data indicates processed DTC samples retain viability and RNA expression profiles, including stress and apoptosis genes, indicating cells are unaltered by instrument processing and amenable to downstream molecular analyses.

## Discussion

Here, we present COSMOS, a platform for the characterization, classification, isolation, and enrichment of cells based on high-dimensional, quantitative morphology profiles. Recent work has motivated image-based cell sorting by using visual cell phenotypes as an analyte but with the prerequisite of using fluorescent labels, which can (1) exclude biomarker-negative but biologically interesting cells in heterogeneous populations for discovery purposes and (2) compromise downstream viability due to chemical toxicity or molecular alterations^14^. Our user-friendly framework enables deep interpretation of single-cell morphology in real-time, without prerequisites for sample pre-processing, cell gating, feature engineering, and/or bioinformatics capabilities, thereby enabling discovery and analysis of cell populations with unknown phenotypic or molecular makeup.

The applications of this platform are similar to other image-based cell sorting methods, including the classification and purification of target cells using visual phenotypes. However, COSMOS extends this technology with several main advances for either research or clinical use, as recently envisioned by other field experts^30,31^. First, by isolating viable unaltered cells from either solid or liquid tissue, sorted cells can be further cultured or grown ex vivo for live functional assays such as 3D organoids, in vitro assays, drug testing to guide therapeutic decisions, and/or inform mechanism of action. Second, although cells are classified in real-time based on complex phenotypes using machine learning, high-content images are captured and stored in the image database. This cloud-enabled image database, which has amassed >1.5 billion images to date, allows for continuous measurement and reanalysis of captured images to potentially detect additional phenotypes resulting from differing cell states/types, drug treatments, or genetic perturbations at single-cell resolution.

One limitation of COSMOS is that it relies on images and sorts single cells flowing in suspension, which requires a dissociation step if applied to solid tissues. The dissociation process could alter the morphology of single cells after tissue dissociation. However, despite this change, we have shown that the potentially altered morphology is still a useful fingerprint to characterize and sort cells of interest within the tissue. A second limitation of COSMOS is its relatively lower throughput in comparison to conventional sorters. This is due to two reasons: (1) the speed of pneumatic valves used for gentle sorting of viable cells and (2) utilization of high-resolution brightfield images and deep inferences, which contrasts with prior related work based on reconstructed images and light computations^13,18–20^. As modeled in Supplementary Fig. 1, depending on desired purity and yield, the sorting throughput of the current system can be pushed to 6000 cells/min. This would be suitable for applications with lower total cell numbers to be processed (hundreds of thousands, up to a million). While we have shown the ability to sort low-abundance cells in this paper (1:100,000), this was done on fixed cells, which allowed flexibility to run longer experiments. If sorting live cells at this rate is desired, the throughput of the current system would be limiting. Addressing the two limiting factors mentioned above could significantly improve the sorting throughout. Third, though the promise of machine intelligence lies in the detection of characteristics indiscernible to the human eye, quantifying and distilling AI-detected distinguishing features as conventionally understandable features, such as size or shape, would enable biological interpretability. Accordingly, future advancements to COSMOS will include the explainability of AI predictions to link machine intelligence to cell biology. Given advances in optics and ultra-high-resolution microscopy, future expansion of this technology could include label-free visualization, classification, and isolation of cells based on subcellular organelle structures and spatial localization. Integration of COSMOS and high-dimensional morphology profiling to current single-cell modalities holds promise in unveiling insights with profound basic, translational, and clinical impact.

## Methods

### COSMOS platform overview

Cells in suspension are input into the cartridge and focused on a single z-plane and lateral trajectory. High contrast, brightfield images of single cells are captured while flowing in the microfluidic cartridge, with two images collected per cell. The model prediction for debris, doublets, and cell clumps is used to report on sample quality, and the softmax layer outputs z-plane focus metrics used to report on image quality. The sorting decision then translates into valve control signals. The laser tracking system detects cells as they arrive at different outlets by evaluating two photomultiplier tube (PMT) signals. The system generates reports of the number and type of analyzed cells, the number of sorted cells, sorting purity and yield, focus plane, synchronization signals, and fluidic pressures and flow rates. The system uses this information in a feedback loop to adjust system parameters.

### Sample processing and cell culture

All human blood samples were collected at external sites according to individual institutional review board (IRB) approved protocols, and informed consent was obtained for each case. For adult control and maternal blood samples, peripheral blood mononuclear cells (PBMCs) were isolated from whole blood by first centrifugation, then the buffy coat was lysed with Red Blood Cell (RBC) Lysis Buffer (Roche) and then washed with PBS (Thermo Fisher Scientific). Fetal cells were isolated from fetal blood by directly lysing with the RBC Lysis Buffer and then washed with PBS. A549, NCI-H1975, NCI-H23 (H23), NCI-H522 (H522), NCI-H810, Hep G2 (HEPG2), SNU-182, SNU-449, SNU-387, Hep 3B2.1–7 (HEP3B2), BxPC-3, PANC-1, Kasumi-1, Reh, and HTR-8/SVneo cell lines were purchased from ATCC and cultured in a humidity and CO_2_-controlled 37 °C cell culture incubator according to ATCC recommended protocols. GM12878 cell line was obtained from the NIGMS Human Genetic Cell Repository at the Coriell Institute for Medical Research and cultured according to their recommended protocols. When applicable, cells were treated with 4% paraformaldehyde (Electron Microscopy Sciences) and stored in PBS at 4 °C for long-term usage of these “fixed” cells. Live cells were used for viability, bulk, and scRNA-Seq experiments.

For experiments in which cell lines were spiked into whole blood, live A549 cells were stained with CellTracker Green CMFDA (Thermo Fisher Scientific), spiked into whole blood (collected in EDTA tubes) at predefined ratios (e.g., 400 or 4000 cells/10 mL blood), followed by buffy coat RBC lysis and fixation. The cell mixtures were pre-enriched by selective depletion of CD45-positive PBMC cells using magnetic beads (Miltenyi Biotec). Then, 20% of each sample was saved for flow cytometry analysis to estimate the number of total cells and cancer cells before and after CD45 depletion. The remaining sample was processed, and A549 cells were sorted on COSMOS.

Dissociated tumor cells (DTCs) from NSCLC patients were purchased from Discovery Life Sciences (DLS; Los Osos, CA, USA). Cancer type, stage information, and cell type composition reports from flow cytometry were provided by the vendor. To account for possible cell type composition changes from the freeze-thaw process, after thawing the DTC aliquots, we split the samples to analyze and sort by flow cytometry. The antibody panel used for flow cytometry included markers for EpCAM, CD45, CD3, CD16, CD19, CD14, and CD11b.

For neutrophil isolation and sorting, human neutrophils were isolated from whole blood using the EasySep Direct Human Neutrophil Isolation kit from Stemcell Technologies by immunomagnetic negative selection. When applicable, isolated neutrophils were labeled with a panel of primary antibodies: anti-CD3, anti-CD45, anti-CD19, anti-CD14, anti-CD66b, anti-CD15 (BioLegend, San Diego, CA) for 20 min at room temperature and washed twice. Live cells (not fixed) were used for experiments, and propidium iodine was added to the cell mixture prior to acquisition and sorting on a BD FACSMelody instrument.

For cell viability assessment, live pre-sorted or sorted cells were stained with either trypan blue or a Calbiochem Live/Dead Double Staining Kit (Millipore Sigma), which uses a cell-permeable green fluorescent Cyto-dye to stain live cells and propidium iodine to stain dead cells. Cells were then counted under a fluorescent microscope.

PBMCs were isolated by Ficoll gradient using Ficoll-Paque (GE Healthcare). CD4+ T cells were isolated using the EasySep™ Human Naïve CD4+T Cell Isolation Kit (Stemcell Technologies) and activated using Dynabeads-conjugated CD3/CD38 human T cell activator (Thermo Fisher Scientific) according to manufacturer instructions and cultured for 3–4 days. Naive or activated T cells were fixed and imaged on COSMOS. For T cell differentiation, cryopreserved naive T cells isolated from PBMCs were purchased from AllCells (Alameda, CA). They were differentiated using the CellxVivo Human Th1 Cell Differentiation Kit protocol (R&D Systems). At timepoints day 0, 1, 2, 3, 4 and 5, cells were collected and imaged live on COSMOS.

### Workflow

A single-cell suspension sample containing 1000–10,000,000 cells (ranging between 5–40 µm in cell diameter) at concentrations up to 500,000 cells/mL was aliquoted into a 15 mL conical tube. For cell-spike experiments, a concentration of 1,000,000 cells/mL was used. The tube containing the sample and a single-use microfluidic cartridge are loaded onto the COSMOS system. On the interface software, several steps are performed to initiate the automated sample run. First, the appropriate pre-trained deep neural network model (e.g., Circulating Cell Classifier) is selected from a drop-down menu of available options, and the desired cell classes are selected for either positive (cell retrieval) or negative (waste) routing outlets. Second, the classifier confidence threshold for identifying and sorting target cell(s) of interest is set according to the desired stringency. Third, the run stop criteria, such as the number of analyzed cells, the number of sorted cells, or the volume of processed sample, is set as desired. From this point on, analysis and sorting are performed automatically by the system without user involvement. The microfluidic cartridge is automatically brought into the field of view of the imaging module, and the z-plane focus is automatically adjusted. The cell suspension sample is then pressurized, and individual cells flow into the microfluidic cartridge. A combination of inertial focusing and sheath flow is used to focus the cells on a specific lateral and z-focusing position into a single file. Images of cells flowing through the microfluidic cartridge are captured and classified based on user selection and then sorted into the positive or negative collection reservoir. After the run is completed, target cells are retrieved from the system. For data analysis, images of all cells run through COSMOS are automatically sent to the Deepcell Cloud, which provides an analysis platform to visualize cell images from specific runs, generate UMAPs of the embedding space, and run additional deep neural network models on samples in silico.

### Microfluidics

Each cartridge design has a microfluidic channel height between 15 and 40 µm, chosen to be a few micrometers greater than the largest cells to be processed. A filter region at the input port prevents large particles, cells or cell aggregates from entering the flow channel. A buffer reagent (1X PBS) is introduced into the flow alongside the cell suspension on either side, achieving hydrodynamic focusing that keeps cells flowing at a consistent speed near the center of the flow horizontally. The flow rate used (~10 µL/min) is also high enough that the effects of inertial focusing^32^ are realized, confining cells to the vicinity of two vertically separated planes close to the center of the flow channel.

### Hardware

A microfluidic cartridge allows for the input and flow of cells in suspension with confinement along a single lateral trajectory to obtain a narrow band of focus across the *z*-axis. Using a combination of hydrodynamic and inertial focusing, we collect high-speed brightfield images of cells (up to 1.2 million frames/minute) as they pass through the imaging zone of the microfluidic cartridge. Images capture subcellular and subnuclear features of the single cells in high contrast, with each pixel representing an area of 0.044 µm^2^. The automated object detection module (see Supplementary Materials) tracks the cells as they flow through the channel. The images are fed into a CNN for the generation of high-dimensional morphological descriptors and classification in real-time. Based on the classification, pneumatic valves are used for sorting a cell into either the cell collection reservoir or waste outlet (Fig. 1a, d and Supplementary Fig. 1a–c). Sorted cells are then retrieved for downstream analysis. A laser-based tracking system identifies cells in real-time to assist with imaging, sorting and reporting on the purity and yield of the run. The instrument can automatically align the microfluidic chip within the camera’s field of view, re-focus the optical z-plane, and adjust its operation based on sensors during instrument setup, imaging, and sorting.

### Cell annotation

Images of single cells are the input to the AI-assisted image annotation software (Fig. 1b), which uses an unsupervised learning approach to assign annotations to images to train machine learning models. Agglomerative clustering is used to cluster cell images, which can be viewed grouped by their focal plane. These cell groups are generated in 2 modes: (1) clusters that are formed based on morphological similarities deduced by an expressive unsupervised model, and (2) morphological proximity to cells annotated within the same session or prior sessions. This software enables a human expert, such as a cytotechnologist, to re-assign annotations to cells that are incorrectly annotated or partition morphologically distinct clusters into multiple cell annotations. Trained users typically annotate up to 6000 cells/minute using this tool. For quality control, multiple rounds of labeling were performed by independent labelers, and when mismatch rates between independent labelers exceeded 5%, runs were queued back to the re-labeling pipeline.

### DCA

The DCA is a database of expert-annotated images of single cells collected from a variety of immortalized cell lines, patient body fluids, and human patient tissue biopsies. At the time of this manuscript, DCA has amassed over 1.6 billion images of single cells. The annotations are structured based on a cell taxonomy which may allow a cell to be assigned multiple annotations on its lineage. The training pipeline extracts training and validation sets from DCA to train and evaluate neural net models aimed at identifying certain cell types and/or states. During training, one or more annotations may be selected for each cell image according to the architecture of the model (Fig. 1c).

### Training and validation sets

Images were split into training and validation sets. Model performance was measured on the validation set. Distinct samples were used for training versus validation to evaluate how well the model generalizes to new samples. Samples were split such that at least 30% of images were used for validation while maximizing sample diversity in the training set. The final models were derived from the TensorFlow implementation for InceptionV3^33^, with custom model training parameters such as image augmentation algorithms that mimic imaging artifacts. Two classifiers were developed and used in this study: Circulating Cell Classifier (Fig. 2, Supplementary Figs. 3, 5, 6, 8, 9 Supplementary Tables 1, 2, 3, 5) and Lung Tumor Classifier (Fig. 3, Supplementary Figs. 4, 10, 11, Supplementary Table 7) as outlined in Supplementary Table 6. For the Circulating Cell Classifier training, out-of-focus cells and debris images constitute 39.01% and 6.57% of the sample, respectively. For the Lung Tumor Classifier, out-of-focus cells and debris images constitute 17.06% and 15.37% of the sample, respectively.

### Model performance

Model accuracy (Figs. 2e, f, 3a, Supplementary Figs. 5a–c, 10a) was measured on in silico mixtures of known cell types from the validation set (Supplementary Table 6). Out-of-focus cells and debris images (Supplementary Fig. 1d) were filtered out from the validation set before measuring performance. The model performance was further biologically validated (Fig. 2g, h) with simultaneous classification and enrichment experiments using spike-in mixtures of known cell types.

### Machine learning

A machine learning infrastructure capable of real-time analysis of cell images was developed to generate high-dimensional morphologic descriptors and classifications (Fig. 1c). Our model architecture is based on the InceptionV3^33^ CNN, modified for grayscale images and to output quantitative morphological descriptors (often called “embeddings” in the machine learning literature). This architecture consists of 48 layers and 24 million parameters. Features from cell images are summarized as an embedding from which cell class annotations are predicted. These embedding vectors are not generally interpretable in terms of conventional morphology metrics but can be used to perform cluster analysis to group morphologically similar cells and visualized using tools like UMAP^34^ and clustered heatmaps. This architecture runs in real-time on our instrument, allowing images to be analyzed by previously trained models and generate classification and high-dimensional morphology descriptions for each imaged cell. When applicable, the model outputs are used to determine whether to discard or retain each cell and, if retained, which collection well to route each cell.

### Brightfield imaging of cells in flow

The microfluidic cartridge is mounted on a stage with lateral (horizontal) XY control and a fine Z control for focus. The objectives, camera, laser optics, and fluidics components are all mounted on the same platform. After the microfluidic cartridge is loaded into COSMOS, it is automatically aligned, and a focusing algorithm is used to bring the imaging region into the field of view. An LED illumination light (SOLA SE) is directed to the imaging region, and multiple images of each cell are captured as it flows through. Brightfield images are taken through objectives of high magnification (Leica 40X–100X) and projected onto an ultra-high-speed camera. To achieve higher accuracies and adjust for potential artifacts in the image, at least two images are captured from each cell as they flow downstream in the channel. These high-resolution cell images reveal not only the cell shape and size but also finer cellular structural features within the cytoplasm and the nucleus that are useful for discriminating cell types and states based on their morphology.

### Computation

The COSMOS software workload is distributed over an Intel Xeon E-2146G central processing unit (CPU), a Xeon 4108 CPU, an Nvidia® Quadro P2000 Graphical Processing Unit (GPU) and a custom microcontroller. The camera is periodically polled for the availability of new images. Image frames from the high-speed brightfield camera are retrieved over a dedicated 1 Gbps ethernet connection. Images are cropped to center cells within them, and the cropped images are sent to the GPU for classification by an optimized CNN that has been trained on relevant cell categories. The network architecture is based on the InceptionV3 model architecture^33^, is implemented using the TensorFlow v1.15^35^ and is trained using cell images annotated with their corresponding cell categories. Nvidia® TensorRT™ (v7.0.0) is used to create an optimized model that is used for inference on the GPU. The classification inference from the models is sent to the microcontroller, which in turn sends switching signals to synchronize the toggling of valves with the arrival of the cell at the sorting location. To maximize throughput, image processing happens in a parallel pipeline such that multiple cells can be in different stages of the pipeline at the same time. The primary use of the GPU is to run the optimized CNN. Some basic image processing tasks, such as cropping cells from the images, are performed on the instrument CPU. The instrument CPU is also used to control all the hardware components and to read in sensor data for monitoring. The training and validation tasks are set up as recurring Apache Beam-based data processing pipelines in the Google Cloud Platform (GCP). Training and prediction jobs are orchestrated by Apache Airflow, and Google Cloud Dataflow is used to combine predictions, embeddings, and annotations. Models are trained using TPU Pod Operators on Google Cloud on version 3 of Google’s Tensor Processing Units. PostgreSQL, Google Big Query, and Google Cloud Storage are used to store and query model predictions, embeddings, and run metadata.

### Data augmentation and model training

Several steps were taken to make the image classifier robust to imaging artifacts by systematically incorporating variations in cell image characteristics into our training data. Cells were imaged under a range of optical focus conditions to sample the effects of changes in focus during and across instrument runs. Images are gathered across four instruments to sample instrument-to-instrument variation. Several augmentation methods were implemented to generate altered replicas of the cell images used to train our classifier. These included standard augmentation techniques such as horizontal and vertical flips of images, orthogonal rotation, the addition of Gaussian noise, and synthetic scaling of intensity contrast variation. We also added salt-and-pepper noise to images to mimic microscopic particles such as dust or other pixel-level aberrations. Finally, we incorporated an additive augmentation to simulate specific noise patterns observed from the camera and framebuffer that are sometimes visible as patterns vertical stripes in some of our early images. Finally, we studied systematic variation in our image characteristics to develop custom augmentation algorithms that simulate chip variability and sample-correlated imaging artifacts on our microfluidic cartridge.

All cell images were resized to 299 × 299 pixels to make them compatible with the InceptionV3 architecture. We trained a model comprising cell types present in normal adult blood, cell types specific to fetal blood, trophoblast cell lines, and multiple cancer cell lines drawn from NSCLC, HCC, pancreatic carcinoma, acute lymphoblastic leukemia (ALL), and AML. The model was also trained to detect out-of-focus, debris, and cell clump images as additional model output classes. This information was used for auto-focusing during instrument runs and to exclude out-of-focus cell images from possible misclassification (Supplementary Fig. 1d).

### AI-assisted annotation of cell images

For the supervised model, we collected high-resolution images from 25.7 million cells, including cells from normal adult blood, fetal blood, trophoblast cell lines, and multiple cell lines derived from NSCLC, HCC, pancreatic carcinoma, ALL, and AML. Images were collected by an ultra-high-speed brightfield camera as cell suspensions flowed through a narrow, straight channel in a microfluidics cartridge. We deployed a combination of techniques in self-supervised, unsupervised, and semi-supervised learning to facilitate cell annotation on this scale. First, we used subject and sample source data to restrict the set of class labels permitted for each cell; as an example, fetal cell class annotations were disallowed in cells drawn from non-pregnant adult subjects. Next, we extracted embedding vectors for each cell image in two pre-trained CNNs: one trained on the ImageNet dataset^36^ and the other on a subset of our own manually annotated cell images. We then used agglomerative clustering of these feature vectors to divide the dataset into morphologically similar clusters, which were presented for manual annotation, thereby facilitating efficient cell annotation at scale.

To further enhance the accuracy of subsequent cell classification, selectively annotated false positive images were identified from the predictions of previously trained models in an iterative manner. Finally, we balanced the classes to be discriminated by feeding the harder examples of more abundant classes inspired by an active learning approach. The hard examples were identified as those that a model trained on a smaller training set had classified incorrectly^37^.

### Reporting summary

Further information on research design is available in the Nature Portfolio Reporting Summary linked to this article.

### Supplementary information

Supplemental Information

Description of Additional Supplementary Files

Supplementary Data 1

Reporting Summary

## Data Availability

Cell images used to generate presented UMAPs and associated embeddings, predictions, and labels are publicly available at https://github.com/deepcell/Salek_2022. Raw and processed RNA-Seq data were deposited in the NCBI Gene Expression Omnibus (GSE241837). All processed whole genome sequencing and targeted mutation data from this study are in Excel spreadsheet format as Supplementary Data 1. All other raw data can be provided upon reasonable request. The following data are publicly available: images used to generate presented UMAPs, embeddings associated with images, predictions associated with the entire set of images for which we calculated predictions and the corresponding models, and labels associated with the entire set of images.

## Code availability

Software code and data to reproduce representative types of analysis and figures are available at https://github.com/deepcell/Salek_2022. These include scripts used to generate ROC curves, confusion matrices, mutation allele counts, and CNV plots.
