# Author Correction: COSMOS: a platform for real-time morphology-based, label-free cell sorting using deep learning

**DOI:** 10.1038/s42003-023-05415-8

**Published:** 2023-10-09

**Authors:** Mahyar Salek, Nianzhen Li, Hou-Pu Chou, Kiran Saini, Andreja Jovic, Kevin B. Jacobs, Chassidy Johnson, Vivian Lu, Esther J. Lee, Christina Chang, Phuc Nguyen, Jeanette Mei, Krishna P. Pant, Amy Y. Wong-Thai, Quillan F. Smith, Stephanie Huang, Ryan Chow, Janifer Cruz, Jeff Walker, Bryan Chan, Thomas J. Musci, Euan A. Ashley, Maddison (Mahdokht) Masaeli

**Affiliations:** 1 Deepcell Inc; 4025 Bohannon Dr., Menlo Park, CA 94025 USA; 2 Department of Medicine, Genetics, & Biomedical Data Science, Stanford University, Stanford, CA USA

**Keywords:** Biotechnology, Machine learning, Cellular imaging

Correction to: *Communications Biology* 10.1038/s42003-023-05325-9, published online 22 September 2023.

The original version of this Article contained an error in Fig. 1d, in which the UMAP graph was blank. The error has been corrected in both the PDF and HTML versions of the Article.

