# Supplementary material for: COSMOS: a platform for real-time morphology-based, label-free cell sorting using deep learning
Source: Commun Biol. 2023 Sep 22;6:971. doi: 10.1038/s42003-023-05325-9 (PMC10516940; doi:10.1038/s42003-023-05325-9)
Supplement: Supplementary file 1 — Supplemental Information [file 42003_2023_5325_MOESM1_ESM.pdf]

Supplementary Materials

COSMOS: A Platform for real-time morphology-based, label-free cell sorting using deep learning

Mahyar Salek[1*], Nianzhen Li[1], Hou-Pu Chou[1], Kiran Saini[1], Andreja Jovic[1], Kevin B. Jacobs[1], Chassidy Johnson[1], Vivian Lu[1], Esther J. Lee[1], Christina Chang[1], Phuc Nguyen[1], Jeanette Mei[1], Krishna P. Pant[1], Amy Y. Wong-Thai[1], Quillan F. Smith[1], Stephanie Huang[1], Ryan Chow[1], Janifer Cruz[1], Jeff Walker[1], Bryan Chan[1], Thomas J. Musci[1], Euan A. Ashley[1,2], Maddison (Mahdokht) Masaeli[1]

[1]Deepcell Inc; 4025 Bohannon Dr., Menlo Park, CA 94025, USA.

[2]Department of Medicine, Genetics, & Biomedical Data Science, Stanford University, Stanford, CA USA.

*Corresponding author. Email: yar@deepcellbio.com

This file includes:

Supplementary Methods
Supplementary Figures 1 to 11
Supplementary Tables 1 to 7

Supplementary Methods

Imaging and cell classification platform. The optical setup consists of an LED illumination light (SOLA SE), lenses and apertures (Thorlabs), 40X-100X objectives (Leica), and high-speed camera (Vision Research). The imaging resolution is up to 5 pixels/µm (at 100X), with 6 µm polystyrene beads (Polysciences) taking up 30 pixels in both x and y dimensions in images captured with COSMOS. The high-speed camera can take images at rates up to 1.2 million frames per minute. Cells are flowed through a microfluidic channel, using a pressure system (SMC) that applies pressure up to 45 psi. This translates to flow velocity of ~0.2 m/s. At this velocity and 2 µs exposure time, the maximum motion blur is 2 pixels at 100X, which is equivalent to 0.4 µm. Multiple images are captured per cell while the cells are flowing in the microfluidic channel. The captured images are processed by a custom designed object detection algorithm, which can detect particles as small as 5 µm in diameter and used to crop the image to a 200X200 pixel image containing the cell. At the 200X200 pixel resolution, the system has a limitation of capturing images of 6,000 cells/minute. The selected pre-trained deep neural network model is then used to classify the image into a predefined set of classes at the desired confidence threshold (between 0 and 1). Latency time for the image analysis and classification pipeline is between 50- 70 ms to classify a single cell. We have built in parallel processing units that can support real-time AI-based classification of ~4,200 cells/minute.

Automated object detection: The system consists of the high-content brightfield imaging setup and two laser sensors. The high-content brightfield imaging is the same setup that generates the cell images for classification and sorting. A built-in processing core performs motion detection by detecting differences between consecutive image frames continuously. When pixel

differences inside a preset region of interest (ROI) exceed a threshold, an image acquisition trigger signal is raised, and multiple image frames are taken and sent to the computers for image processing and classification. Lasers are laid on the positive and negative sorting channels after the bifurcation point. The laser signals are digitized by a data acquisition module (NI-DAQ) and processed by the CPU through a low-pass filtering and peak detection algorithm. Particles passing through laser spots are detected. Sorting time variations were calculated and delay time between image acquisition and sorting action was fed back to the system to maintain imaging consistency and high sorting yield and purity.

Cell sorting and performance. Cell sorting is performed using built-in pneumatic microvalves on both the positive (cell retrieval) and negative (waste) sides of the flow channel downstream of the bifurcation point. Valve timing is controlled by a digital signal processing (DSP)-based microcontroller circuit with 0.1ms time precision. When the model infers that a cell belongs to a targeted category, switching signals are timed to synchronize the toggling of valves with the arrival of the cell at the flow bifurcation point, and the cell flows into a reservoir on the microfluidic cartridge where targeted cells are collected (positive well). If the model infers that a cell does not belong to a targeted category, the cell flows into a waste tube. Elliptical laser beams are focused onto both the positive and negative output channels downstream of the sorting flow bifurcation to detect passing cells and thereby monitor sorting performance in real-time.

The overall performance of COSMOS cell sorting has two contributing factors: classifier accuracy in identifying a target cell and the microvalves accuracy to divert the cell of interest into a collection reservoir. We evaluated the performance of the cell sorter, specifically as it

relates to the pneumatic valves, excluding the accuracy of the AI classification. To this aim, we ran multiple controlled sorting experiments, where target cells to sort were selected randomly at a certain frequency. The sorting performance was evaluated using our optical tracking system.

We used homogeneous cell suspensions, which were prepared at a concentration of 1,000,000 PBMCs/mL. Each sample was introduced into the microfluidic cartridge at a flow rate of ~2.2 µl/min which corresponds to a throughput of 2,160 cells/minute. A side reagent of 1X PBS buffer was simultaneously introduced to help focus the cells to a single stream. The total flow rate of sample and side reagent is ~10µL/min A fraction (0.5%) of the cells imaged in flow were randomly selected to be sorted into the positive well of the microfluidics cartridge. Laser spots downstream of the bifurcation junction on either side were used to mark the passage of cells and thereby count true positive (TP), false positive (FP) and false negative (FN) sorting events. In each experiment, 50 cells out of a total of ~10,000 imaged cells were selected for sorting, and the yield (sensitivity or recall) and purity (precision or PPV) metrics were calculated as TP/(TP + FN) and TP/(TP + FP) respectively.

There is a trade-off between yield and purity that can be modulated by the valve window size, which is the time window in which flow is diverted toward the positive well for each sorting event. Yield and purity metrics for four different window sizes collected from 341 experimental runs are shown in **Supplementary Figure 1**. For each window size, data were collected from at least 77 independent runs, distributed across 21 microfluidic cartridges, 7 photoresist mold sets and 2 instruments. The cell flow rate affects the number of false positives observed at any given window size and thus influences purity. The yield is not affected by the false positive rate (FPR)

and thus primarily depends on the window size. The results of this characterization are shown in **Supplementary Figure 1**. The theoretical (estimated) curves for yield are created using the least square fit with two fitting parameters, standard deviation (σ) of a normal distribution of cell arrival time and a detection probability (p), i.e. $yield = p * \mathrm{erf}(WindowSize/(2\sigma*\sqrt{2}))$. The best fit is obtained when $\sigma = 5.07$ ms and $p = 93\%$. For purity, the estimation is calculated based on $purity = TPR/(TPR+FPR)$, where *TPR*, true positive rate, is the true yield calculated above by compensating the detection lost, and *FPR* is obtained using the least square fit with two fitting parameters, cell rate (*Rc*) and a minimum cell-to-cell separation (*ts*) in time, a phenomenon known in inertia focusing. i.e., $FPR = Rc * (WindowSize-2*ts)$. The best fit is obtained when $Rc = 35.91$ cells/s and $ts = 3.01$ ms. Even though these two fitting parameters are calculated mathematically, they do agree with real observations. The 341 experiments were conducted by targeting the cell rate at 36 cells/s, matching the estimated number. Theoretical curves are added to show the expected effect of changes in cell flow rate on purity, based on a normally distributed transit time for the cells with a standard deviation of 5 ms. The measured purity is closely consistent with theoretical expectation, while the yield is about 7% lower than expected. As a representative example, these results indicate that with a window size of 25 ms and a flow rate of 2,160 cells/min, the sorted cells for a rare component that constitutes 0.5% of the cells would have a yield of about 90% and a purity of about 60%. The experiments in this work were done at the rate of 2,160 cells/min.The measured data shows consistency in sorting performance across multiple microfluidic cartridges, instruments and runs. At a given number of cells of interest to analyze within an hour, one can adjust valve parameters (**Supplementary Figure 1**) to achieve desirable purity vs yield. While the sorting rate can go as high as 4,200 cells/minute

(which is the limitation on real-time classification), we chose to run experiments in this study at a lower rate to achieve higher purity and yield.

Molecular analyses

Single-cell RNA sequencing (scRNA-Seq). Live DTCs, PBMCs and isolated neutrophils were used in scRNA-Seq experiments. Cells were either directly loaded or retrieved from the positive wells of the microfluidic cartridge following sorting on COSMOS then loaded on a BD Rhapsody single cell analysis system (BD Biosciences, CA). Single cells were then processed following either targeted RNA sequencing (human immune response panel) or whole transcriptome amplification protocols. The sequencing data were analyzed using the BD^{TM} Data View v1.2.2 software (BD Biosciences, CA) software.

Bulk RNA sequencing. Total RNA was extracted from live cells using the RNeasy Mini Kit (Qiagen). cDNA synthesis, amplification and library preparation were performed with the Quantseq 3'm RNA-Seq Library Prep Kit (Lexogen) according to the manufacturer's protocol. The final libraries were sequenced on an Illumina Miniseq. Read QC, trimming, alignment and counting were performed with the Lexogen Quantseq analysis pipeline. Differential expression analysis was done using DESeq2 and iDEP (http://bioinformatics.sdstate.edu/idep/).

Genotyping. Cell lines and PBMCs of individual blood donors were genotyped with Next Generation Sequencing using a targeted SampleID panel (Swift Biosciences) that includes 95 assays for exonic single nucleotide polymorphisms (SNPs) and 9 assays for gender ID. Briefly, genomic DNA was extracted from bulk cells using QIAGEN DNeasy Blood & Tissue Kit

(Qiagen) and 1 ng DNA was used as input to amplify the amplicon panels and prepare the sequencing library. For cancer cell lines, a 20-amplicon panel that covers the full length of the *TP53* gene (Swift Biosciences) was pooled with the SampleID panel so cells were genotyped on both common SNPs and *TP53* mutational status. From ATCC and COSMIC annotation, A549 cells are known to be *TP53* wild type and NCI-H522 are known to carry a homozygous frameshift mutation (c.572_572delC). Our bulk genotyping results confirmed the relative mutation status for these two cell lines. For sorted cells from the COSMOS experiments, cells were retrieved from the positive outlet well of the microfluidic cartridge into a PCR tube, then directly lysed using Extracta DNA Prep for PCR (Quanta Bio). Cell lysates were amplified with the Swift Biosciences amplicon panels and followed by the same library preparation procedure for NGS.

DTCs from lung cancer patients. Cells were analyzed for targeted DNA mutations and copy number variations (CNV) before and after sorting on COSMOS. For mutation analysis following direct lysis with Extracta DNA Prep for PCR (Quanta Bio) a 208-amplicon panel that includes 17 lung cancer genes (Swift Biosciences) were used. For CNV analysis, after direct lysis, genomic DNA was amplified using ResolveDNA Whole Genome Amplification Kit (BioSkryb Genomics) and then libraries were prepared for sequencing (Kapa Hyperplus Kit, Roche). All libraries were sequenced on either an Illumina MiniSeq or NextSeq instrument (Illumina) using 2x150 bp kit (DNA) or 2x75 bp kit (RNA).

Primary sequencing analysis and QC. Sequencing reads were aligned to the reference genome using the BWA-MEM aligner. SNP allele counts were summarized using bcftools. SNP data

were subjected to quality control checks: each sample was required to have a mean coverage per SNP of > 200; each SNP locus needed to have a median coverage across all samples > 0.1x the median SNP to be considered; each individual SNP assay for a sample needed to have a depth of coverage > 50. 89 SNP assays were selected on this basis for further use in mixture analysis. Samples and individual SNP assays that failed QC were excluded from genotyping and the estimation of mixture proportions.

Mixture proportion estimation by SNP analysis. Pure diploid samples that formed the base of each mixture for spike-in experiments were clustered into the three diploid genotypes (AA, AB, BB) for each SNP using a maximum likelihood estimation that incorporated an internal estimate of error within homozygous SN. The mixture proportion of the component of interest (tumor cell line or fetal sample) was determined using maximum likelihood estimation (MLE), in which all discrete mixture fractions in increments of 0.005 were considered (0.0, 0.005, 0.01, …, 1.0). For each possible mixture proportion, expected allele fractions at each SNP were determined by linearly combining the allele fractions in the two mixture components. A binomial log likelihood corresponding to each individual sample-SNP combination was computed using the expected allele fraction and an effective number of independent reads N per SNP estimated from the variance of allele fraction in mixture SNPs at which the base genotype is heterozygous (AB) and the spike-in component genotype is homozygous (AA or BB). By estimating N from the mixture data directly and using SNPs expected to have a shared allele fraction, the procedure is robust to low input for which the number of reads might exceed the number of independent molecules sampled. The overall log likelihood for each possible mixture proportion is computed as the sum of contributions from each SNP, and the mixture proportion is estimated as that at which the

highest overall log likelihood is obtained. The accuracy of the procedure was verified on DNA mixtures with known composition (**Supplementary Figure 7**). Each composite sample contained 250 pg of DNA and the mixture proportion of DNA from the second individual was set at 5%, 10%, 20%, 30%, 40%, 60%, 80%, and 90%.

Joint Estimation of Genotypes and Sample Purity. In two cases, genotypes and mixture fraction were jointly estimated from the allele fractions ϕ of SNPs in the mixture: (i) to genotype the fetal sample Fet1, which included some maternal cells in addition to fetal cells (ii) for the spike-in of A549 cells into whole blood. In each case, genotypes for one of the mixture components, designated $\boldsymbol{G_0}$, were obtained from a pure sample (from maternal DNA for the former, and from the pure A549 cell line for the latter), while the genotypes of the other sample, designated $\boldsymbol{G}$ (corresponding to the fetal sample in the former case and to the unrelated blood sample for the latter) were estimated from the data. The maternal sample was genotyped as diploid, but for pure A549, the allowed allele fractions for genotypes were 0, ⅓, ½, ⅔ and 1, in keeping with the known hypotriploidy of that cell line. An expectation maximization (EM) procedure was then used to jointly estimate the purity and missing genotypes. Briefly, given $\boldsymbol{G_0}$ and a current estimate of purity f, a binomial likelihood was estimated for each allowed missing genotype, and a maximum likelihood estimate was used to update $\boldsymbol{G}$. Given $\boldsymbol{G}$, a revised estimate of f was obtained by linear regression, using the expected linear relationship between the observed allele fraction ϕ and $\boldsymbol{G_0}$ over SNPs of identical $\boldsymbol{G}$. The procedure incorporated an error rate estimate drawn from the SNPs where both components are identically homozygous. The procedure was iterated until convergence, defined as changes in the purity estimate < 0.0001.

Mutation and CNV analysis. Mutation allele fractions in sorted samples were estimated from targeted amplicon sequencing data. In each sample, the mutation allele fraction was estimated as the fraction of high-quality read alignments overlapping the mutation locus that contained the variant allele. For spike-in samples, the pre-enrichment allele fraction is the spike-in concentration used (1:1000, 1:10,000 or 1:100,000).

Six aliquots from the GM12878 cell line, consisting of 100, 50, 25, 10, 5 and 1 cell(s) respectively, were used as a normalization cohort for copy number estimation in DTCs before and after enrichment. Read coverage was first aggregated over 1 Mb genomic intervals across the genome within the dissociated tumor sample and each of the GM12878 normalization aliquots. The coverage within each sample was then scaled by the mean coverage per Mb over the entire genome for that sample. Next, the median assay bias and median absolute deviation (MAD) of the scaled coverage for each 1 Mb interval across the genome were computed from data from the normalization cohort. Genomic intervals for which the MAD across normalization samples exceeded 20% of the median were excluded from further analysis. Finally, the coverage values within the dissociated tumor sample before and after enrichment were further scaled by the median assay bias estimated from the normalization cohort.

Software and algorithms

Cell annotation for model training purposes. Our proprietary annotation software contains a command-line tool for clustering and annotating cell images with cell class labels. It can either annotate cells from a single run or it can accept a list of cells to which to assign labels from a JSON file. The tool tracks annotation progress locally and writes annotations to Google Cloud

Storage. Labeling of cell images is optimized by clustering cells with similar embeddings, so that panels of cells can be displayed with similar morphology and are more likely to have uniform labels.

Model training. Our proprietary model training software contains tools for training and validating models.

- **/trainer/configs:** Configuration files used in defining model construction, training, evaluation, and scheduling on GCP.
- **/scripts:** Scripts to test and run various tools: automatic labeling, filtering of debris and out of focus cells, start TensorRT, tuning jobs, etc.
- Customizing cell taxonomy: It allows to customize the class taxonomy for a given model, e.g., asserting that WBC is parent class for T-cell classes
- Monitoring in-progress models is done via tensorboard service (https://www.tensorflow.org/tensorboard).
- A CI/CD Pipeline automatically tests and deploys changes to a private repository and a private NPM repository.

Run initialization software.

- Cartridge auto-aligner: scans the cartridge for the visual markers and it moves the stage to the accurate spot for imaging
- Cartridge auto-focuser: scans the cartridge for the visual markers and it refocuses the camera based on predetermined optimal focus pattern with the visual markers

Imaging and classification.

- Cell detector and cropper: Runs an algorithm based on running averages to identify background vs foreground thresholds. It then applies standard computer vision algorithms based on border following[1] to identify cell contour and use growing and shrinking region techniques to crop each cell from the image background.
- Image normalizer: Normalizes the image by mapping the darkest pixel to 0 and the brightest to 255 and linearly interpolates the values in between.
- Object tracker: Multiple images of the same cell are grouped together via an object tracking algorithm that estimates flow trajectories. Typically, each cell will have two images captured.
- Cell classifier: Inputs each cropped and normalized cell image through a deep learning model via tensorRT server to classify images in real time. An ensemble consensus algorithm combines classifications from each image of a cell and generates a consensus classification in two modes: average prediction based on image-based predictions and voting.

Cell sorting.

- Valve control module that operates the timing and actuation of valves on sorting channels
- Laser detection and timing module that detects passing of cells under laser spots and calculates velocity and estimated time of arrivals for cells.
- Run metric evaluator: monitors various run metrics such as flow rate and pressure and throws warning and exceptions if they are out of bound.

Real-time adjustment.

- Flow adjuster: A PID controller to keep the flow values within the range.
- Real-time z-plane focuser: A feedback algorithm based on real-time calculation of focus scores and the direction of out-of-focus. The out of focus indications are a result of the trained neural network.

Supplementary Figures

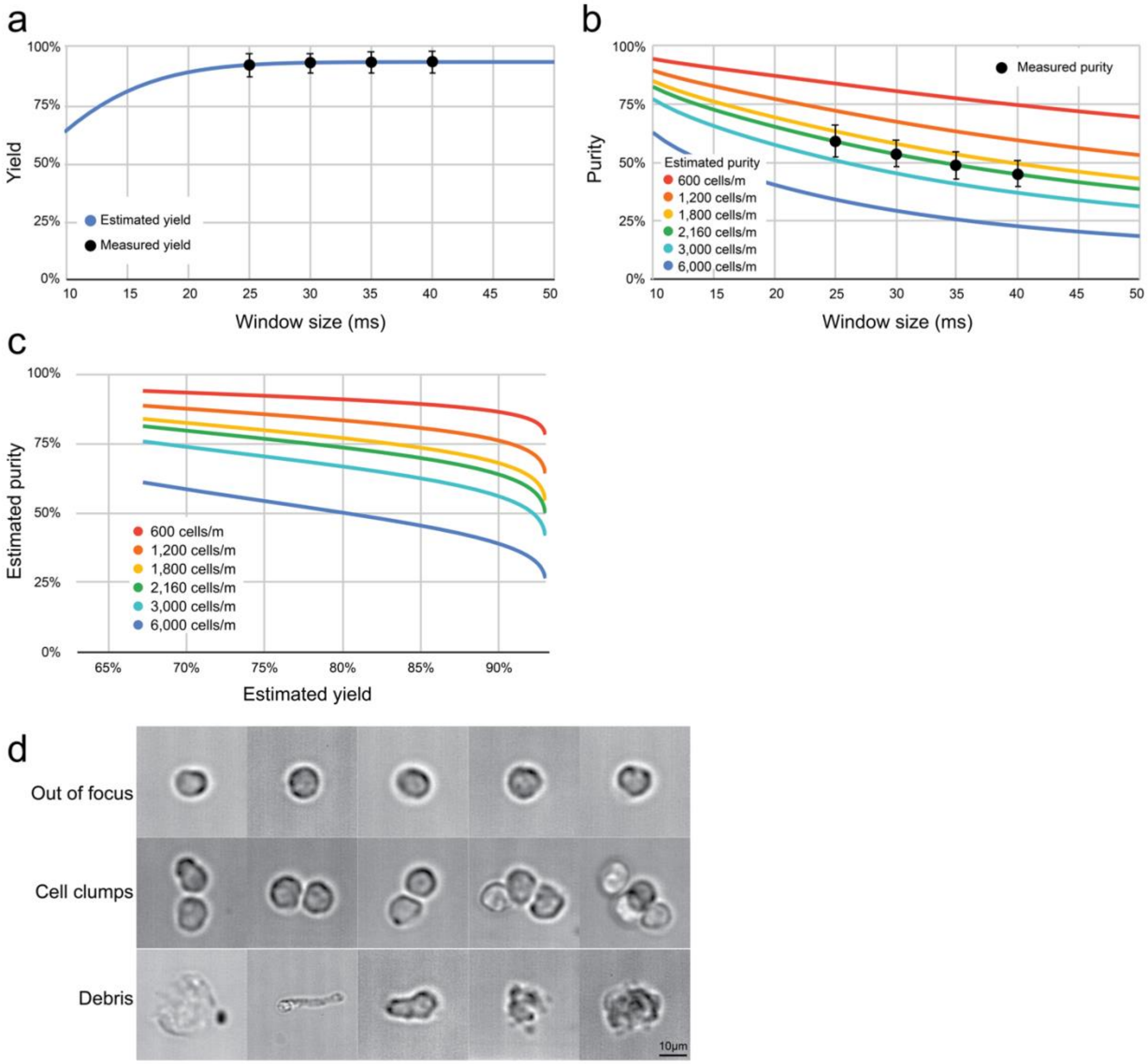

Supplementary Figure 1. Performance of cell sorting under 0.5% random sorting of PBMC samples using different window sizes (25, 30, 35 and 40 milliseconds). A total of 341 experiments were run across four window sizes in 21 microfluidic devices (three chips each from seven photoresist mold sets) and on two hardware systems. (**a**) Yield: The fitted curve is created using the least square fit with two fitting parameters, standard deviation of a normal distribution of cell arrival time (σ) and a detection probability (p), i.e. $yield = p * \mathrm{erf}(WindowSize/(2\sigma*\sqrt{2}))$. The best fit is obtained when $\sigma = 5.07$ ms and $p = 93\%$. (**b**) Purity: $purity = TPR/(TPR+FPR)$, where *TPR*, true positive rate, is the true yield calculated above by compensating the detection lost, and *FPR*, false positive rate, is obtained using the least square fit with two fitting parameters, cell rate (*Rc*) and a minimum cell-to-cell separation in time (*ts*), a phenomenon known in inertia focusing. i.e. $FPR = Rc * (WindowSize\text{-}2*ts)$. The best fit is obtained when $Rc = 35.91$ cells/s and $ts = 3.01$ ms. Even though these two fitting parameters are calculated mathematically, they do agree with real observations. The 341 experiments were conducted by targeting the cell rate at 36 cells/s, perfectly matching the fitted number. (**c**) Purity to yield trade-off curve for different cell rates. (**d**) Representative images of low-quality images, which are trained and validated as their own class. Low quality images are filtered out before measuring performance. Scale bar as indicated. Error bars in graphs **a** and **b** represent one standard deviation (± one sigma) of the raw experimental data in each window size.

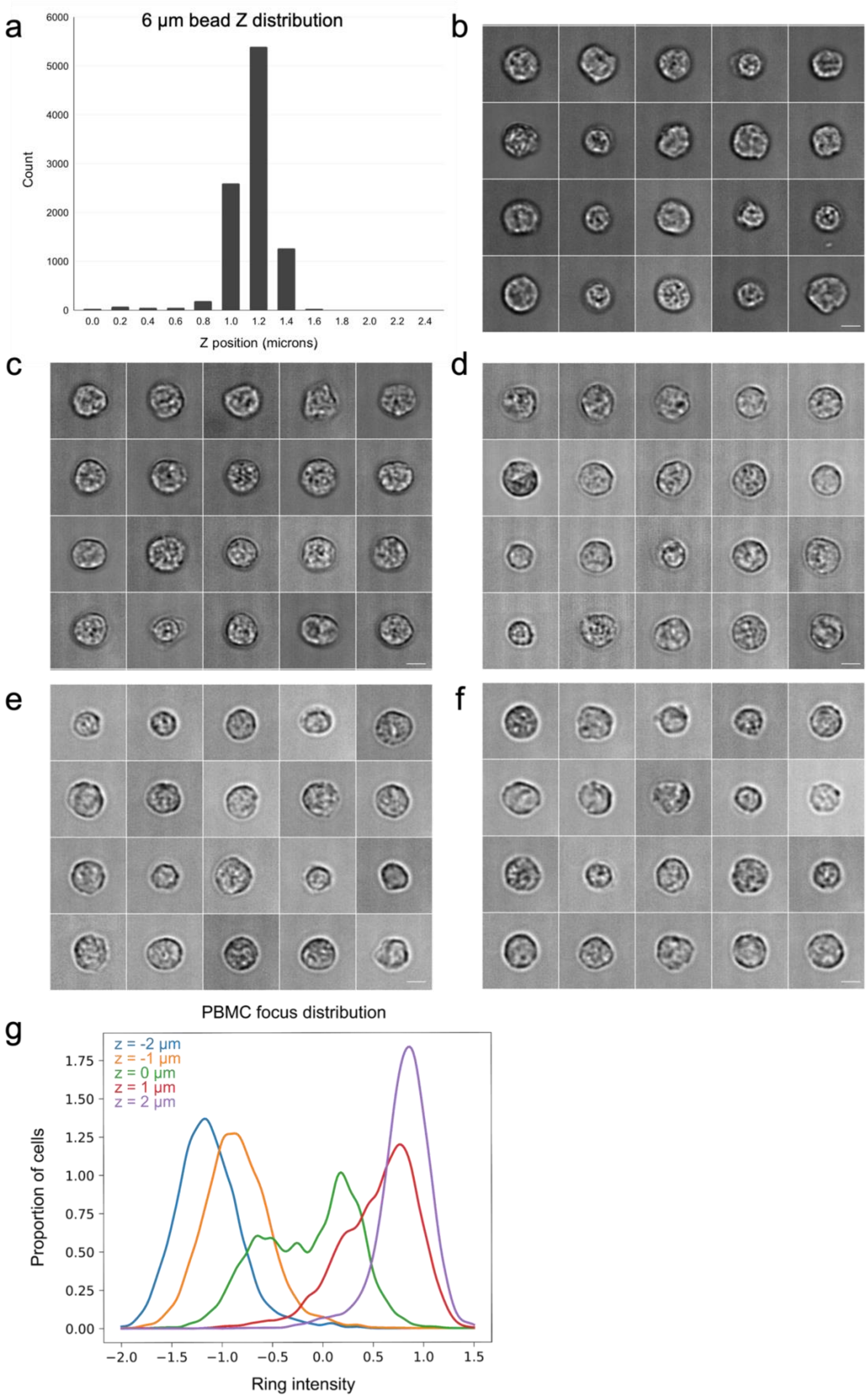

a
6 μm bead Z distribution
Count
Z position (microns)
b
c
d
e
f
g
PBMC focus distribution
z = -2 μm
z = -1 μm
z = 0 μm
z = 1 μm
z = 2 μm
Proportion of cells
Ring intensity

Supplementary Figure 2. Ultra-right focus range. (a) Distribution of Z positions of 9,698 6-µm polystyrene beads in a single imaging run: standard deviation is 0.21 µm. The Z positions were calculated from a calibrated mapping from bead appearance (values such as gradient strength at the edge of the beads) and controlled Z stacks of bead images. **(b)-(f)** Images of fixed human PBMC cells flowing through a microfluidic channel. The offset from the focal plane to the flow plane are **(b)** -2, **(c)** -1, **(d)** 0, **(e)** +1, **(f)** +2 µm. Visual inspection shows >95% cells are within +/- 1 µm, which is consistent with (g) quantification of ring intensity distribution. Scale bar indicates 5µm.

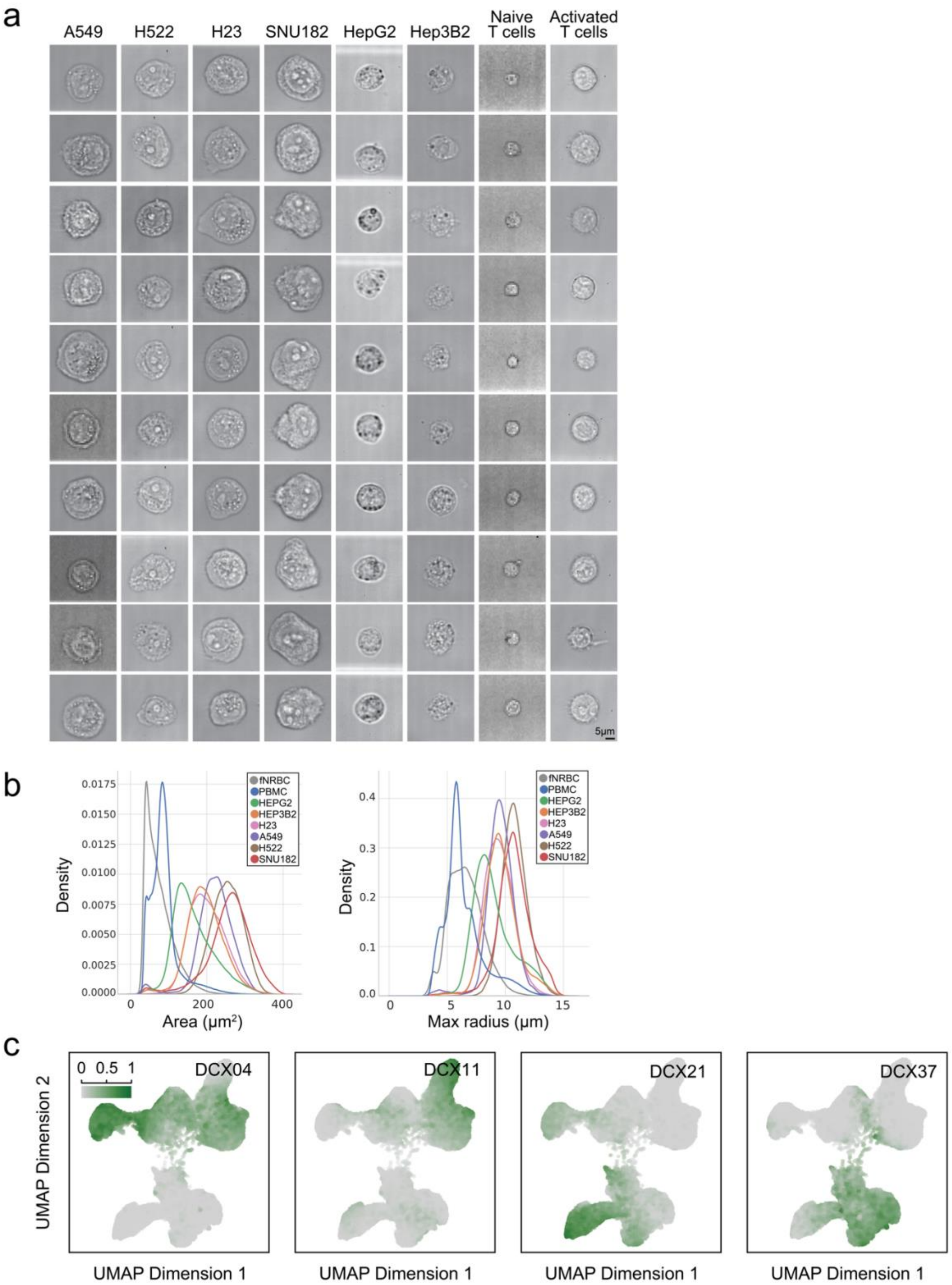
a
A549
H522
H23
SNU182
HepG2
Hep3B2
Naive T cells
Activated T cells
5μm
b
Density
Area (μm²)
Max radius (μm)
fNRBC
PBMC
HEPG2
HEP3B2
H23
A549
H522
SNU182
c
0 0.5 1
DCX04
DCX11
DCX21
DCX37
UMAP Dimension 2
UMAP Dimension 1

Supplementary Figure 3. Cell size and embedding dimension of cells. **(a)** Representative images of A549, H522, H23, SNU182, HepG2, Hep3B2, naïve T cells, and activated T cells (related to **Figure 2b**). Scale bar as indicated. **(b)** Area and maximum radius measurements of the indicated cells. **(c)** UMAP projection of the indicated coordinate in the embedding space of the model. The value of the coordinate (arbitrary unit: 0-1) is represented by the heatmap color demonstrating the contribution of that coordinate in identifying cells that are highlighted. For example, the value of coordinate number 4 (DCX04) shows that this coordinate "encodes" for the NSCLC and HCC (malignant) cells, whereas coordinates DCX11, DCX21, and DCX37 correspond to the HCC, fnRBC, and PBMC classes, respectively.

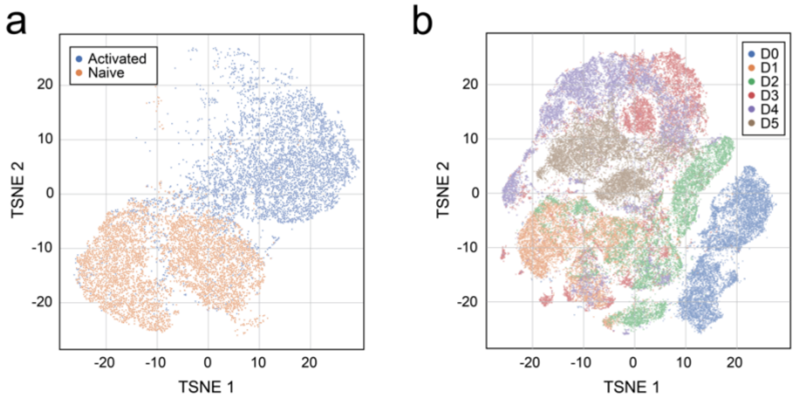
a
Activated
Naive
TSNE 2
TSNE 1
b
D0
D1
D2
D3
D4
D5
TSNE 2
TSNE 1

Supplementary Figure 4. tSNE projection of cell embeddings for T cell states. (a) Naive and Dynabead CD3/CD28 activated CD4+ T cells and (**b**) a time course (day 0, 1, 2, 3, 4, and 5) differentiation study of CD4+ T cells were imaged on COSMOS and the cell embeddings were plotted by tSNE analysis. Each dot represents a single cell.

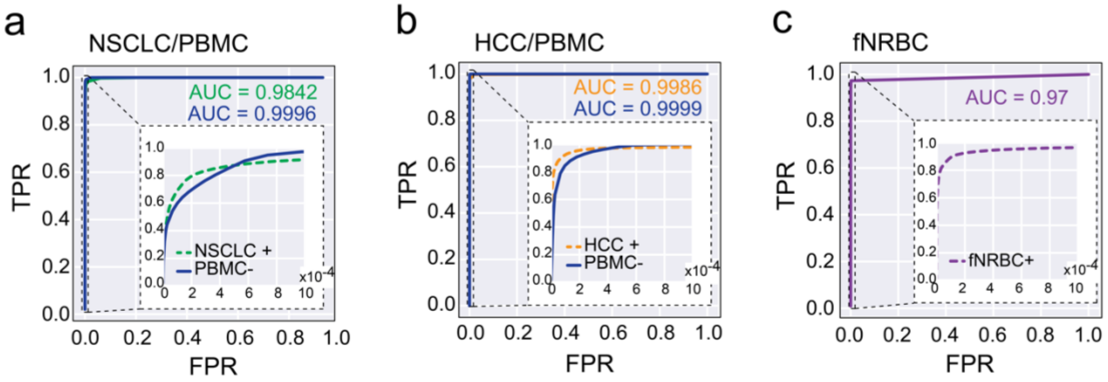
a
NSCLC/PBMC
AUC = 0.9842
AUC = 0.9996
NSCLC +
PBMC-
TPR
FPR
b
HCC/PBMC
AUC = 0.9986
AUC = 0.9999
HCC +
PBMC-
TPR
FPR
c
fNRBC
AUC = 0.97
fNRBC+
TPR
FPR

Supplementary Figure 5. Receiver operating characteristic (ROC) curves for classification. **(a)** NSCLCs and **(b)** HCCs are shown. Two ROC curves each are shown: one for the positive selection of each category, and one for negative selection, specifically for the selection of non-blood cells. Area Under Curves (AUCs) achieved for NSCLC are 0.9842 (positive selection) and 0.9996 (negative selection) and for HCC are 0.9986 (positive selection) and 0.9999 (negative selection). (**c**) ROC curves for the classification of fnRBCs and the AUC is 0.97 (positive selection). Insets zoom into the upper left portions of the ROC curves where false positive rates are very low to highlight the differences between modes of classification.

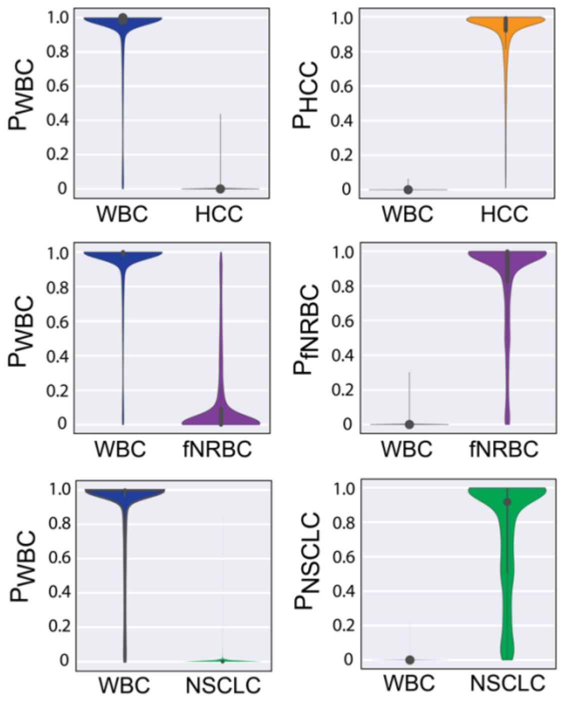

P_{WBC}
1.0
0.8
0.6
0.4
0.2
0
WBC
HCC
P_{HCC}
WBC
HCC
P_{WBC}
WBC
fNRBC
P_{fNRBC}
WBC
fNRBC
P_{WBC}
WBC
NSCLC
P_{NSCLC}
WBC
NSCLC

Supplementary Figure 6. Violin plots showing the predicted probabilities and data distribution of assigning cells in each category to its appropriate class. WBCs (P_{WBC}), HCC (P_{HCC}), fNRBC (P_{fNRBC}), and NSCLC (P_{NSCLC}) are shown.

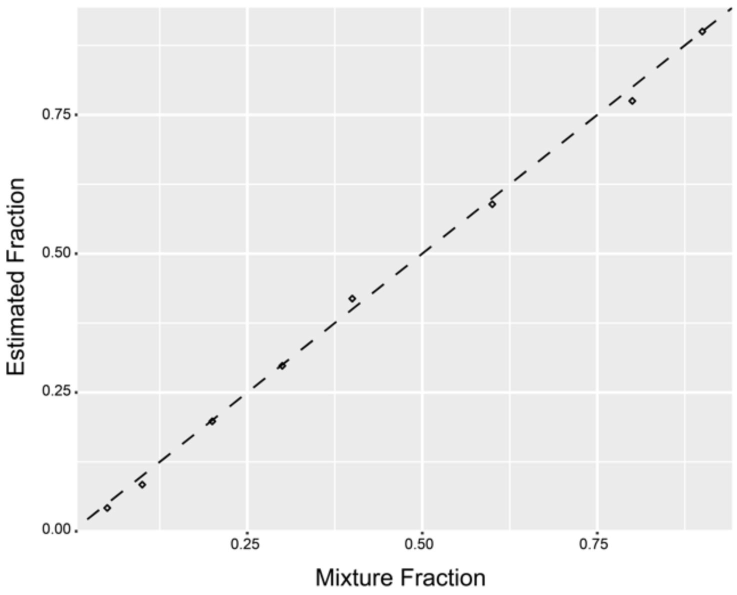
Estimated Fraction
0.75
0.50
0.25
0.00
0.25
0.50
0.75
Mixture Fraction

Supplementary Figure 7. Accuracy of SNP-based mixture fraction estimates in control DNA mixtures. Each composite sample contained 250 pg of bulk DNA drawn from two individuals and the mixture proportion of DNA from the second individual was set at 5%, 10%, 20%, 30%, 40%, 60%, 80% and 90%. A close correspondence was found between the known and estimated mixture proportions.

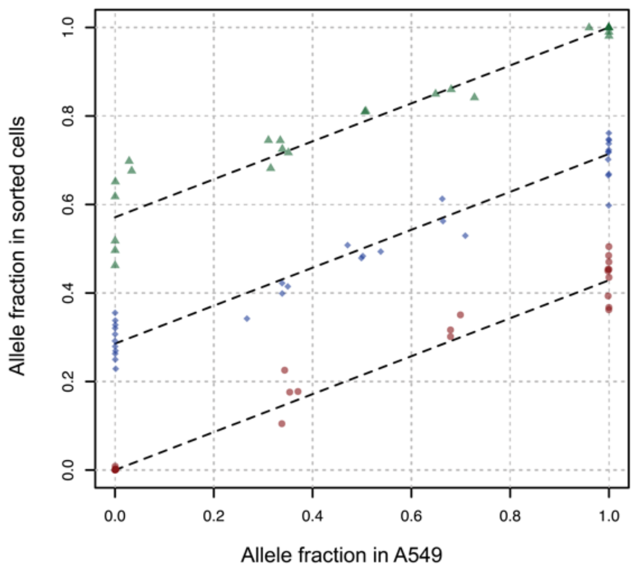

Allele fraction in sorted cells
Allele fraction in A549
0.0
0.2
0.4
0.6
0.8
1.0

Supplementary Figure 8. Purity of spiked A549 cells in COSMOS enriched samples. A549 cells were spiked at 40 cells/mL into healthy donor whole blood. The purity and blood sample genotypes were estimated with an expectation-maximization (EM) algorithm. Red circles, blue diamonds and green triangles denote AA, AB, and BB genotypes respectively in the blood sample used as a base for the spike-in mixture. Dotted lines depict the linear regression used to estimate the purity given the genotypes; their slope is equal to the final purity estimate of 0.43 (95% confidence interval 0.40 - 0.45).

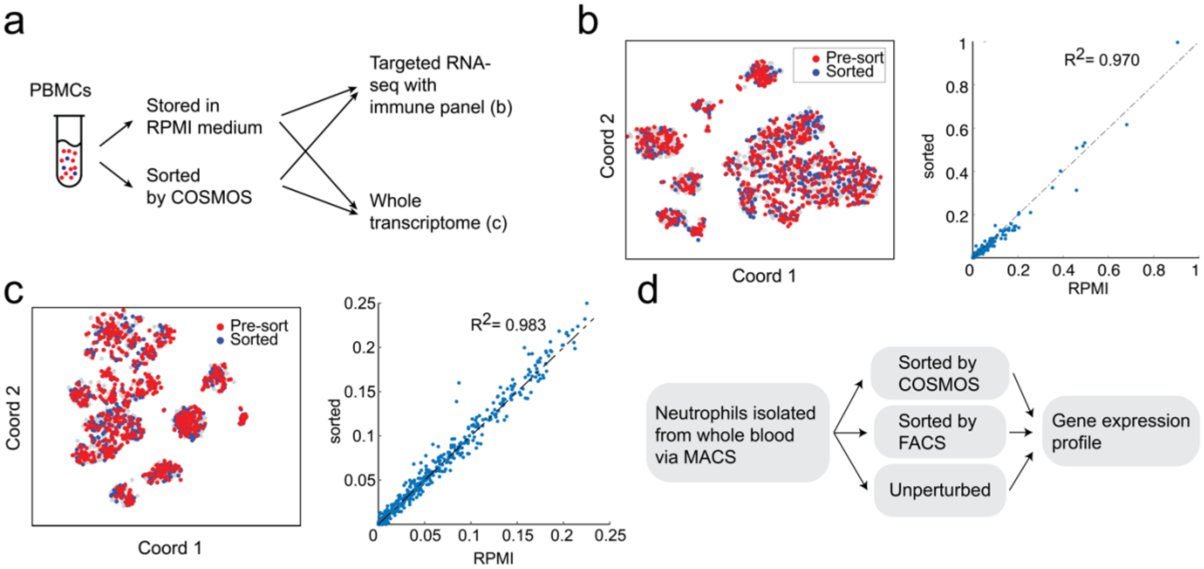

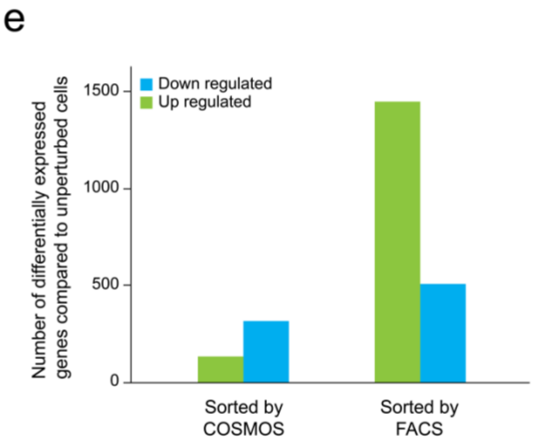

f

adj.Pval	nGenes	Pathways
7.45E-12	49	Myeloid cell activation involved in immune response
7.45E-12	56	Leukocyte activation involved in immune response
7.45E-12	49	Leukocyte degranulation
1.39E-11	46	Neutrophil mediated immunity
2.04E-11	45	Neutrophil degranulation
2.13E-11	75	Immune effector process
2.13E-11	45	Neutrophil activation involved in immune response
2.13E-11	46	Granulocyte activation

Supplementary Figure 9. Minimal gene expression changes in COSMOS processed PBMCs and neutrophils. (**a**) Workflow schematic: PBMCs imaged on COSMOS and collected (sorted) were compared with control cells stored in RPMI medium using either a single cell targeted immune panel (**b**) or single cell whole transcriptome (WTA) workflow (**c**) with the BD Rhapsody™ system. (**b** and **c**) t-SNE plots of gene expression profiles of the pre-sort and sorted cells, each point is a cell. Corresponding correlation plots of mean (log_{10}(molecules per cell per gene)) for the two conditions, each point is a gene. The two samples overlapped with each other in t-SNE plots and gene expression levels showed high correlations (R^2 equaled 0.97 and 0.98 for targeted panel and WTA, respectively), indicating no significant change in gene expression after sorting. (**d**) Workflow schematic: human neutrophils were first isolated from whole blood by immunomagnetic negative selection then split into multiple aliquots for four conditions: unperturbed (untreated), immunostained, and negative selection sorted by FACS, unstained/unlabeled and sorted by COSMOS. Pre-sorted and sorted cells were lysed for bulk gene expression profiling by RNA-Seq. (**e**) The number of differentially expressed genes compared to unperturbed cells as determined by bulk RNA-Seq for COSMOS and FACS sorted samples are summarized. COSMOS sorted neutrophils had a lower number of up- and down-regulated genes compared to FACS confirming that COSMOS-sorted cells had minimal gene expression differences compared to unperturbed cells. (**f**) A summary of the upregulated pathways in FACS-sorted compared to COSMOS-sorted neutrophils indicating FACS, but not COSMOS, induced upregulation of pathways in neutrophil activation and degranulation.

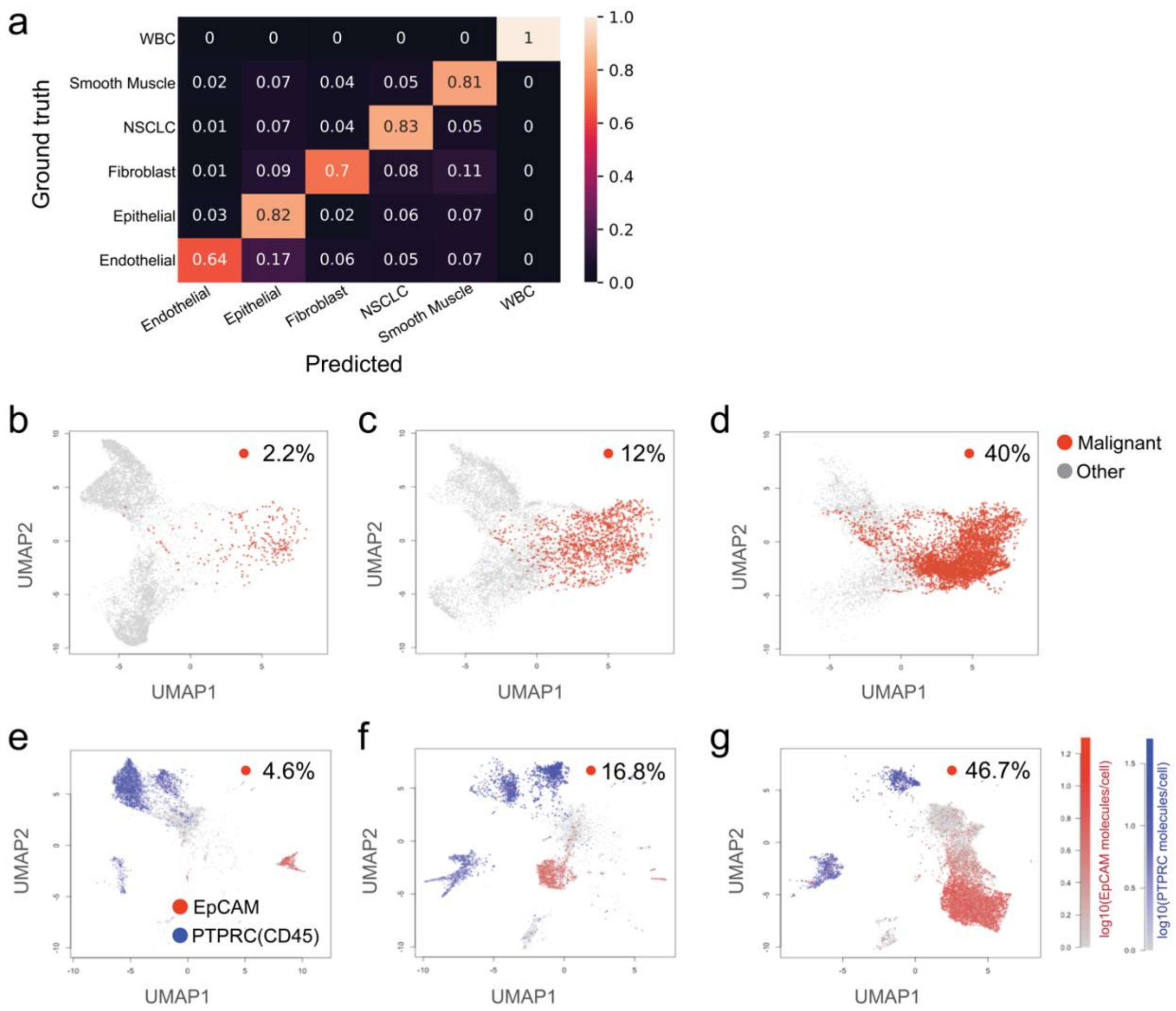
a
Ground truth
Predicted
WBC
Smooth Muscle
NSCLC
Fibroblast
Epithelial
Endothelial
Endothelial
Epithelial
Fibroblast
NSCLC
Smooth Muscle
WBC
0 0 0 0 0 1
0.02 0.07 0.04 0.05 0.81 0
0.01 0.07 0.04 0.83 0.05 0
0.01 0.09 0.7 0.08 0.11 0
0.03 0.82 0.02 0.06 0.07 0
0.64 0.17 0.06 0.05 0.07 0
1.0
0.8
0.6
0.4
0.2
0.0
b
c
d
2.2%
12%
40%
Malignant
Other
UMAP1
UMAP2
e
f
g
4.6%
16.8%
46.7%
EpCAM
PTPRC(CD45)
log10(EpCAM molecules/cell)
log10(PTPRC molecules/cell)

Supplementary Figure 10. Morphology-based deep learning model predicted a similar fraction of malignant vs nonmalignant cells as scRNA-Seq. (**a**) Confusion matrix representing Lung Cancer Classifier prediction accuracy (x-axis) vs the ground truth (y-axis) on training dataset. Individual components including endothelial, fibroblasts, smooth muscle, and epithelial cells are shown. Morphology UMAPs of embeddings and scRNA-Seq gene expression of three different dissociated tumor cell (DTC) NSCLC samples containing low (**b, e**), medium (**c, f**) and high (**d, g**) percentage of malignant cells were tested. (**b-d**) UMAP of morphological embeddings: each data point is a cell; the predicted malignant cells are colored red and non-malignant cells colored gray. The plot labels indicate the fraction of malignant cells predicted by the model. (**e-g**) scRNA-Seq gene expression UMAP for all genes. The red and blue color gradients indicate the expression levels of EpCAM (tumor cell marker) and PTPRC (CD45, immune cell marker), respectively. The plot labels indicate the fraction of EpCAM+/PTRPC- cells. scRNA-Seq and morphology UMAPs have similar resolution and separation of malignant vs nonmalignant cells for all three samples.

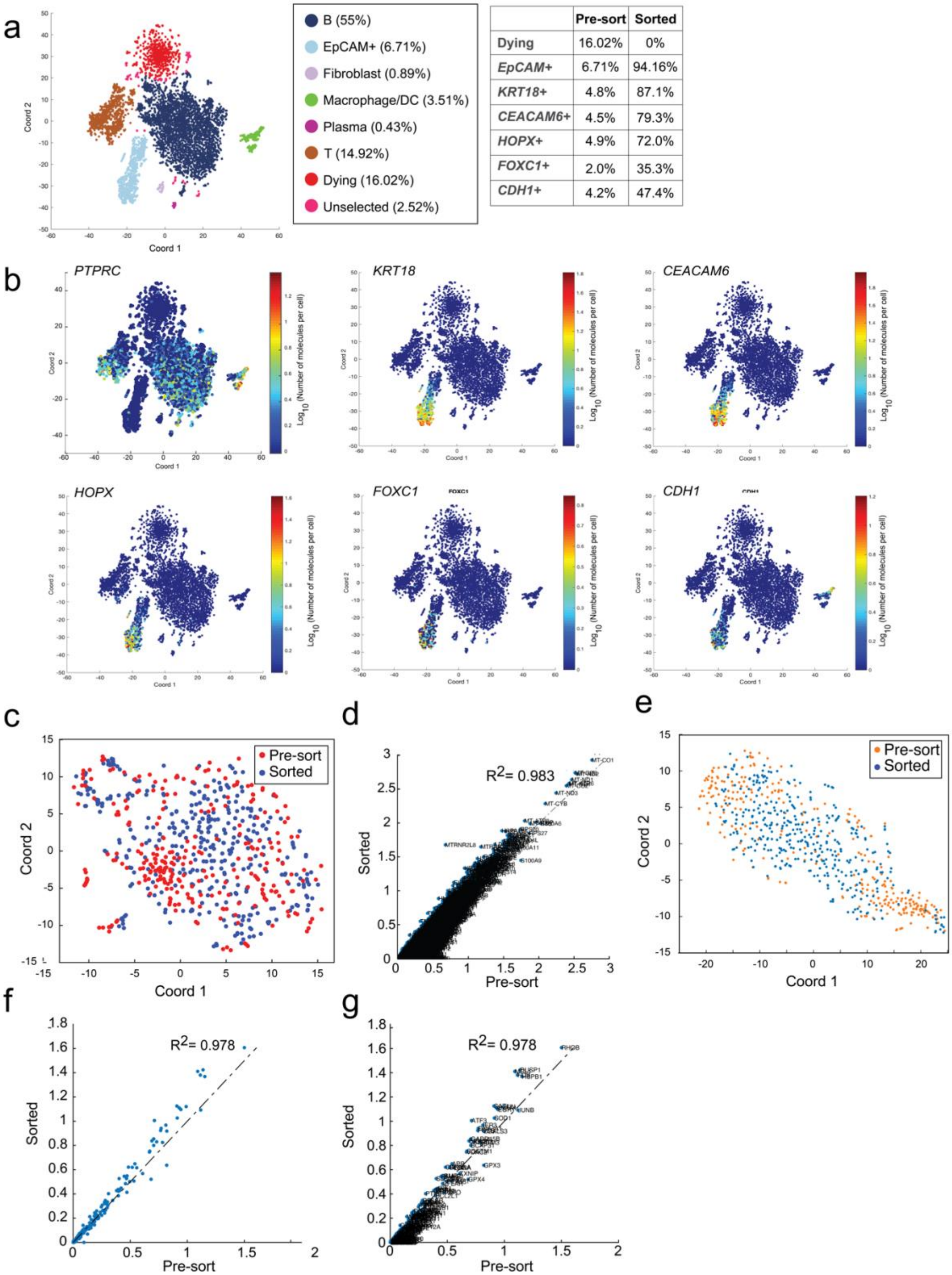

	Pre-sort	Sorted
Dying	16.02%	0%
EpCAM+	6.71%	94.16%
KRT18+	4.8%	87.1%
CEACAM6+	4.5%	79.3%
HOPX+	4.9%	72.0%
FOXC1+	2.0%	35.3%
CDH1+	4.2%	47.4%

Supplementary Figure 11. scRNA-Seq profiling of DTCs. (**a**) UMAP of scRNA-Seq gene expression of a DTC sample with annotated cell clusters and corresponding percentages of each cell class (**b**) Pseudo-color gene expression levels of immune cell marker (*PTPRC*, encoding CD45) and NSCLC markers (*KRT18*, *CEACAM6*, *HOPX*, *FOXC1*, *CDH1*) of DTC sample (related to **Fig. 3h**). (**c**) Further sub-clustering of the EPCAM$^+$/PTPRC(CD45)$^-$ cluster showed that sorted cells almost completely overlapped with pre-sorted cells for all subclusters. (**d**) Gene expression correlation plot of mean ($\log_{10}$(molecules per cell per gene)) for the sorted and the pre-sorted cells from the EPCAM$^+$/PTPRC(CD45)$^-$ cluster from **Fig. 3i** with the gene names annotated. (**e-g**) Stress and apoptosis related gene expression profile comparison of the pre-sorted and sorted cells in the EPCAM+/CD45- subpopulation. (**e**) Further sub-clustering of the EpCAM$^+$/CD45$^-$ cluster from **Fig. 3i** using only the 166 stress and apoptosis genes, showed sorted cells overlap with pre-sort cells in all subclusters and. **(f** and **g)** Gene expression correlation plots with gene names annotated is shown. Each data point is a gene and the names annotated. The gene expression correlation coefficient (R^2) was 0.978 suggesting that COSMOS sorting did not cause additional cell stress.

Supplementary Table 1. Cell spike into PBMCs and SNP analysis to estimate enrichment and purity of cells processed on COSMOS.

Cell Line	Cell Type	Spike-in ratio	No. cells imaged	Event rate (images/min)	Classifier positive rate	No. classifier positive cells	Estimated sorted cell purity	Estimated fold enrichment	Run duration (hr)
Fet1	fnRBC	1:1,304	999,978	1089.6	0.017%	172	74%	965	15.30
A549	NSCLC	1:1,000	69,611	730.2	0.147%	103	62%	348	1.59
A549	NSCLC	1:1,000	101,180	1369.8	0.172%	174	67%	380	1.23
A549	NSCLC	1:10,000	1,105,997	1292.4	0.045%	492	27%	1,978	14.26
A549	NSCLC	1:10,000	876,421	1035.6	0.082%	723	17%	1,201	14.15
A549	NSCLC	1:10,000	1,107,669	1344.6	0.025%	279	31%	2,305	13.73
A549	NSCLC	1:10,000	1,063,745	1140.6	0.0083%	88	42%	4,200	15.55
A549	NSCLC	1:10,000	1,169,744	1161.6	0.0094%	110	33%	3,300	16.80
A549	NSCLC	1:10,000	719,499	1321.2	0.028%	200	33%	3,300	9.08
H522	NSCLC	1:10,000	1,050,036	1076.4	0.030%	314	26%	2,550	16.26
A549	NSCLC	1:100,000	1,342,632	1136.4	0.003%	43	20%	13,904	19.69
H522	NSCLC	1:100,000	1,514,263	1428	0.0045%	72	30%	30,000	17.67
H522	NSCLC	1:100,000	1,561,847	1320	0.006%	94	33%	32,500	19.72

Supplementary Table 2. Cells spiked into PBMCs and genotyping analysis using known frameshift mutations to estimate enrichment and purity of cells processed on COSMOS.

Cell Line	Spike-in ratio	Proportion of TP53 frameshift c.572_572delC	Fold enrichment of c.572_572delC
A549	100%	0%	--
NCI-H522	100%	100%	--
NCI-H522	0.1% (1:1,000)	45%	451
NCI-H522	0.01% (1:10,000)	15%	1,499
NCI-H522	0.001% (1:100,000)	23%	22,770

Supplementary Table 3. SNP analysis to estimate enrichment and purity of A549 cells spiked into whole blood.

Spike-in (cells/ml)	%A549 after RBC lysis	Post-CD45 Depletion		Cells imaged	Event rate (image/ min)	Classifier		Sorted cell purity	Fold enrichment	Run duration (hr)
		%A549	Fold enrichment			Positive Rate	Positive cells			
400/mL	0.004%	0.060%	13	1,045,016	1210.8	0.019%	196	55%	10,900	14.39
400/mL	0.003%	0.060%	16.2	932,665	902.4	0.018%	164	80%	29,000	17.22
40/mL	0.001%	0.010%	11	969311	973.2	0.007%	70	43%	33,500	16.60
40/mL	0.001%	0.010%	6.7	1,012,315	1063.8	0.009%	94	35%	27,800	15.86

Supplementary Table 4. Cell viability after processing on COSMOS. All runs were between 60-120 minutes.

Sample	No. runs	Cells imaged	Event rate range (images/min)	Cell concentration range (cells/mL)	% Viability of pre-sorted cells (average ± SD)	% Viability of sorted or flown-through cells (average ± SD)
PBMC	13	9709390	1200 - 27720	1e6 - 30e6	94.7 ± 2.5%	94.2 ± 3.8%
B-lymphoblastoid cell line (GM12878)	10	1056618	1200 - 1500	1e6	90.6 ± 3.1%	91.0 ± 5.4%
Cancer cell lines (H522 and A549)	13	10609835	1200 - 22548	1e6 - 30e6	96.1 ± 2.0%	96.9 ± 2.2%
Dissociated NSCLC tissue	3	323410	1200 - 1548	1e6	71.0 ± 4.4%	65.0 ± 1.0%

Supplementary Table 5. Full list of enriched pathways in FACS-sorted vs COSMOS-sorted neutrophils.

Adjusted p-value	Number of Genes	Pathways	Genes
1.54E-04	47	Symbiont process	LTF THOC5 EIF3D CALM2 CALM3 CAMP PCBP1 JUN APOBEC3G CCL3 MPO PSMB1 MRE11 EIF2AK2 WAPL TNPO1 KPNA3 SGTA FKBP8 DDX58 VPS29 LTBR NFE2L2 CD46 VPS4B IRF3 BECN1 ILF3 TRIM22 TANK TAF1 ZYX ITGA5 SYK LMBRD1 RAB6A SATB1 KPNA4 NUCKS1 RAB1B PTBP1 SERPINB9 AP2S1 RPL21 RPS4Y1 NMT2 POLR2A
1.41E-03	41	Viral process	EIF3D PCBP1 JUN APOBEC3G CCL3 PSMB1 MRE11 EIF2AK2 WAPL TNPO1 KPNA3 SGTA FKBP8 DDX58 VPS29 LTBR NFE2L2 CD46 VPS4B IRF3 BECN1 ILF3 TRIM22 TANK TAF1 ZYX ITGA5 SYK LMBRD1 RAB6A SATB1 KPNA4 NUCKS1 RAB1B PTBP1 LTF AP2S1 RPL21 RPS4Y1 NMT2 POLR2A
1.41E-03	75	Cellular response to stress	MRE11 MAP2K4 UBE2A ACD SGTA LTBR NPRL2 NFE2L2 PRDX6 RPA2 BECN1 HSPA2 RBM17 ERCC5 HUS1 OXSR1 TMEM259 UBE2G2 ZNF652 TAF1 SIRT6 GSK3B SUSD6 GADD45G POLN RHOB OPA1 SMC5 MPO BID EIF2AK2 TSPO CINP CCDC47 COL4A3BP SLC12A4 MAX DYSF TANK PARP9 PEA15 SYK ATMIN TERF2IP DDIT4 NABP1 JUN CALR UPP1 ERO1A IRAK4 AGER SIPA1 CCDC88C PDK3 NUCKS1 THOC5 CBX3 MCM7 FEM1B PTTG1IP KMT5A CHUK ARHGEF6 PSMB1 TAB2 SH2D3C CAMKK2 IRF3 CNOT6L TPP1 DNAJC7 YIF1A POLR2A GFPT1
1.52E-03	93	Protein localization	TMSB10 TBC1D22A ZC3H11A WAPL TNPO3 RABL2B TNPO1 SUN2 THOC5 TM9SF1 ACD STX10 SGTA KDELR1 VPS29 TM9SF2 BECN1 KIF13A SRP14 SRP9 IMMP1L COMMD1 RAB1B ZDHHC14 RAB6A SVBP EXOC3 PTTG1IP IPO9 BID BTN3A1 TOLLIP GSK3B TESC DVL1 SRSF3 RPA2 APPL2 FLCN CALR CD24 MYCBP2 LSG1 AP2S1 DGKD BIRC5 SNAP29 TSPO KPNA3 CORO1C VPS4B FFAR1 IRF3 KTN1 YWHAQ DYSF CTDSPL2 ARL3 ARL8A RHOB FRMD4A AHCTF1 GHRL FCHO2 ZFAND2B CALM3 SYK GAPVD1 FNTA YIF1A SMAD2 RABEP2 KPNA4 AGER SNX2 DENND1B SFT2D2 PARP9 SCIN YBX1 CD33 DDX58 TRIM22 TERF2IP UBE2G2 CHUK CCL3 GNAS CASC3 RPL21 RPS4Y1 HAX1 ATP6V1B2
1.52E-03	73	Protein transport	TBC1D22A ZC3H11A TNPO3 RABL2B TNPO1 THOC5 STX10 SGTA VPS29 KIF13A SRP14 SRP9 IMMP1L RAB1B ZDHHC14 RAB6A SVBP PTTG1IP IPO9 BTN3A1 GSK3B SRSF3 APPL2 CALR CD24 BID LSG1 AP2S1 DGKD TESC BIRC5 SNAP29 KPNA3 KDELR1 VPS4B FFAR1 IRF3 KTN1 YWHAQ DYSF CTDSPL2 ARL3 ARL8A RHOB FRMD4A AHCTF1 GHRL ZFAND2B SYK GAPVD1 COMMD1 YIF1A SMAD2 RABEP2 EXOC3 KPNA4 AGER SNX2 DENND1B SFT2D2 ACD CD33 DDX58 UBE2G2 CHUK CCL3 GNAS TSPO CASC3 RPL21 RPS4Y1 HAX1 ATP6V1B2
1.52E-03	75	Amide transport	TBC1D22A ZC3H11A TNPO3 RABL2B TNPO1 THOC5 STX10 SGTA VPS29 COL4A3BP KIF13A SRP14 SRP9 IMMP1L GHRL SLC19A1 RAB1B ZDHHC14 RAB6A SVBP PTTG1IP IPO9 BTN3A1 GSK3B SRSF3 APPL2 CALR CD24 BID LSG1 AP2S1 DGKD TESC BIRC5 SNAP29 KPNA3 KDELR1 VPS4B FFAR1 IRF3 KTN1 YWHAQ DYSF CTDSPL2

			ARL3 ARL8A RHOB FRMD4A AHCTF1 ZFAND2B SYK GAPVD1 COMMD1 YIF1A SMAD2 RABEP2 EXOC3 KPNA4 AGER SNX2 DENND1B SFT2D2 ACD CD33 DDX58 UBE2G2 CHUK CCL3 GNAS TSPO CASC3 RPL21 RPS4Y1 HAX1 ATP6V1B2
1.60E-03	67	Intracellular transport	GSK3B CCDC88C TBC1D22A ZC3H11A TNPO3 RABL2B TNPO1 SNAP29 THOC5 STX10 SGTA KDELR1 VPS29 COL4A3BP BECN1 KIF1C KIF13A SRP14 SRP9 IMMP1L SYK RAB1B ZDHHC14 RAB6A PTTG1IP IPO9 SNX2 DENND1B ATP5MG MT-ATP8 LSG1 CORO1C SRSF3 VPS4B APPL2 RHOB TMCC1 CALR CD24 BID AP2S1 SUN2 KPNA3 DVL1 TFG RAP1B YWHAQ CTDSPL2 ARL3 ARL8A ZFAND2B CALM3 KPNA4 SRSF10 ACD UBE2G2 MYO10 GNAS HMGXB4 TSPO CASC3 RPL21 WDR60 RPS4Y1 ACTR10 HAX1 OPA1
2.05E-03	73	Peptide transport	TBC1D22A ZC3H11A TNPO3 RABL2B TNPO1 THOC5 STX10 SGTA VPS29 KIF13A SRP14 SRP9 IMMP1L GHRL RAB1B ZDHHC14 RAB6A SVBP PTTG1IP IPO9 BTN3A1 GSK3B SRSF3 APPL2 CALR CD24 BID LSG1 AP2S1 DGKD TESC BIRC5 SNAP29 KPNA3 KDELR1 VPS4B FFAR1 IRF3 KTN1 YWHAQ DYSF CTDSPL2 ARL3 ARL8A RHOB FRMD4A AHCTF1 ZFAND2B SYK GAPVD1 COMMD1 YIF1A SMAD2 RABEP2 EXOC3 KPNA4 AGER SNX2 DENND1B SFT2D2 ACD CD33 DDX58 UBE2G2 CHUK CCL3 GNAS TSPO CASC3 RPL21 RPS4Y1 HAX1 ATP6V1B2
2.05E-03	75	Establishment of protein localization	TBC1D22A ZC3H11A TNPO3 RABL2B TNPO1 THOC5 STX10 SGTA VPS29 KIF13A SRP14 SRP9 IMMP1L RAB1B ZDHHC14 RAB6A SVBP PTTG1IP IPO9 BID BTN3A1 GSK3B SRSF3 APPL2 CALR CD24 LSG1 AP2S1 DGKD TESC BIRC5 SNAP29 TSPO KPNA3 KDELR1 CORO1C VPS4B FFAR1 IRF3 KTN1 YWHAQ DYSF CTDSPL2 ARL3 ARL8A RHOB FRMD4A AHCTF1 GHRL ZFAND2B CALM3 SYK GAPVD1 COMMD1 YIF1A SMAD2 RABEP2 EXOC3 KPNA4 AGER SNX2 DENND1B SFT2D2 ACD CD33 DDX58 UBE2G2 CHUK CCL3 GNAS CASC3 RPL21 RPS4Y1 HAX1 ATP6V1B2
2.08E-03	29	Small GTPase mediated signal transduction	RABL2B GNA13 RHOF RHOB RAB1B RAB6A ARL3 JUN CCDC125 DENND4B RFXANK SH2D3C SOS2 DOCK8 RALGPS2 RAP1B ARPP19 ARHGEF6 YWHAQ BNIP2 VAV1 TIAM2 DOCK11 KCTD13 CHUK SIPA1 FLCN ARHGAP25 SIAH2
2.11E-03	76	Establishment of localization in cell	GSK3B CCDC88C TBC1D22A ZC3H11A TNPO3 RABL2B TNPO1 SNAP29 THOC5 STX10 SGTA KDELR1 VPS29 COL4A3BP BECN1 KIF1C KIF13A PLCB2 SRP14 SRP9 IMMP1L FCHO2 SYK RAB1B ZDHHC14 RAB6A PTTG1IP IPO9 SNX2 DENND1B CALM2 CALM3 ATP5MG MT-ATP8 LSG1 CORO1C SRSF3 VPS4B APPL2 RHOB TMCC1 CALR CD24 CCL3 BID AP2S1 BIRC5 SUN2 KPNA3 DVL1 TFG RAP1B YWHAQ CTDSPL2 ARL3 ARL8A GHRL ZFAND2B MEI1 KPNA4 SRSF10 ERO1A ACD CEP19 UBE2G2 MYO10 GNAS HMGXB4 TSPO CASC3 RPL21 WDR60 RPS4Y1 ACTR10 HAX1 OPA1
2.54E-03	96	Negative regulation of metabolic process	HIVEP1 PHF12 ATAD2B ARPP19 FXR2 CARHSP1 REL SERPINB9 JUN SVBP PTBP1 LTF EIF2AK2 SMARCA2 AES SGTA DVL1 TRIM37 CSRNP2 NPRL2 CD46 TSHZ3 CBX3 MAX ILF3 YWHAQ TUT4 TAF1 MGAT5 FLCN PHF6 GHRL TERF2IP ZBTB33 CALR KMT5A SRSF10 ARID5A CCL3 MYCBP2 YBX1 SIRT6 TCF7 TESC BIRC5 MAEA TSPO CHD8 PHF14 CASC3 BIRC6 NMI IRF3 BECN1 MLLT1 TRIM22 AKIRIN2 MICAL1 DYSF APPL2 HUS1 CNOT6L SRP9 YY1AP1 RYBP DDIT4 ESRRA SMAD2 CEP295NL SIAH2 RNF41 SATB1 RBM10 CEBPD ACD MRE11 WAPL FKBP8 CORO1C PARP9 FAM192A ANGEL2

			UBE2G2 TFE3 GSK3B JAK3 PSMB1 TAB2 TNPO1 RPL21 RPS4Y1 CALM2 CALM3 RBBP4 S100A11 POLR2A
2.54E-03	30	Myeloid leukocyte mediated immunity	SYK CCL3 DDX58 SERPINB9 IRAK4 MPO PSMB1 LTF CAPN1 ATP11A TOLLIP SNAP29 ASAH1 CD33 PDAP1 BST1 PRDX6 CYSTM1 RAP1B ACTR10 RHOF SRP14 C1orf35 ARL8A SURF4 EEF1A1 S100A11 CAMP OSCAR RAB6A
2.71E-03	66	Regulation of intracellular signal transduction	TFG BID MAP2K4 LTBR NPRL2 GADD45G IRAK4 CCL3 LTF GNAS TRIM22 CALM2 MGAT5 CALM3 RNF41 CCDC125 DENND4B CD24 EIF2AK2 SOS2 RALGPS2 IRF3 BECN1 RAP1B ARPP19 ARHGEF6 BNIP2 VAV1 TIAM2 FLCN SYK TERF2IP DDIT4 KCTD13 JUN CALR AGER SIPA1 TAB2 REL OPA1 PTBP1 GSK3B BST1 NFE2L2 TANK PARP9 HAX1 GHRL ARHGAP25 PTTG1IP KMT5A CHUK MRE11 TAF2 PPP2R5C JAK3 RPA2 GNA13 HUS1 RHOF RHOB TAF1 RBBP4 PEA15 TAF15
2.79E-11	83	Intracellular transport	NPEPPS PICALM RAB7A SAR1A RAB2A SEC61B IFT20 MAK SNX17 TMED5 SPCS3 NAPG BLOC1S1 STX11 RBM26 RHOT2 DOP1B HSPD1 RAB5A GOLGA7 RABGAP1L RANBP2 ZDHHC7 STX5 ZDHHC3 TBC1D10C FES BCAP31 POM121 CHMP1B EPS15 MAVS ATXN1 ZC3H12A RNF139 IFIT1 CHP1 DDX39B PDCD6 LAMP2 CD74 CDC42 CBL ERBIN HSPA9 RAP1A TXN MAPK8IP3 LAMTOR1 RILPL2 PAN3 SRP68 CREB3L2 HGS FAM160A2 GSK3A AUP1 PEX16 OSBPL2 AKT1 DPY30 TSC1 LGALS9 ARHGAP1 PTPN1 TAP2 VAMP2 ACAA1 CAPZB MAPK3 RPL34 PPP6C RPL36 HMGA1 EIF4A3 DYNC1LI1 FIP1L1 RPS3A DDX19B ACOX1 RPL9 RPS26 RPL23A
2.79E-11	93	Establishme nt of localization in cell	NPEPPS PICALM RAB7A SAR1A RAB2A SEC61B IFT20 MAK SNX17 TMED5 SPCS3 NAPG BLOC1S1 STX11 KIAA1109 RBM26 RHOT2 DOP1B HSPD1 RAB5A GOLGA7 RABGAP1L RANBP2 ZDHHC7 STX5 ZDHHC3 TBC1D10C FES BCAP31 POM121 CHMP1B EPS15 MAVS ATXN1 ZC3H12A RNF139 IFIT1 CHP1 DDX39B PDCD6 LAMP2 CD74 BAK1 CDC42 CBL ERBIN HSPA9 ITGA4 RAP1A CANX PPT1 TXN CDK5RAP2 MAPK8IP3 LAMTOR1 RILPL2 PAN3 SRP68 SLC30A1 CREB3L2 ACTG1 HGS NSFL1C UBXN2B FAM160A2 GSK3A AUP1 PEX16 OSBPL2 AKT1 DPY30 TSC1 LGALS9 ARHGAP1 PTPN1 TAP2 VAMP2 ACAA1 CAPZB MAPK3 RPL34 PPP6C RPL36 HMGA1 EIF4A3 DYNC1LI1 FIP1L1 RPS3A DDX19B ACOX1 RPL9 RPS26 RPL23A
5.61E-10	86	Vesicle-mediated transport	CDC42 PICALM RAB7A SAR1A SNX17 TMED5 PPT1 BLOC1S1 STX11 KIAA1109 MAPK8IP3 DOP1B RAB5A GOLGA7 C9orf72 VPS8 XKR8 STX5 FES BCAP31 CHMP1B EPS15 CEACAM1 CSK RNF139 PTPN1 PYCARD RAB2A RAPGEF1 CBL ITGA4 RAP1A MAPK8IP1 CANX NAPG LAMTOR1 RABGAP1L PI4K2A ELMO1 CDC42SE2 CD14 CREB3L2 ACTG1 CHP1 AIF1 VAMP2 FAM160A2 NOTCH1 DPY30 LGALS9 ARHGAP1 TBC1D10C HGS PDCD6 GABARAPL2 LAMP2 CD44 ACAA1 CAPZB MAGT1 MAPK3 MAN2B1 GCA QPCT PPP6C ARAP3 RAP2C ARPC1B JCHAIN CHI3L1 PRCP ADAM10 DYNC1LI1 FAM49B CDA APEH FPR1 LRG1 ATP6AP2 PSMD13 ATP6V0C XRCC6 TAP2 TMSB4X PLEKHO2 MGAM
4.05E-09	56	Intracellular protein transport	NPEPPS RAB7A SAR1A RAB2A SEC61B SNX17 TMED5 SPCS3 NAPG STX11 HSPD1 RAB5A GOLGA7 RABGAP1L RANBP2 ZDHHC7 STX5 ZDHHC3 TBC1D10C POM121 MAVS ZC3H12A IFIT1 BCAP31 CHP1 DDX39B PDCD6 LAMP2 CD74 CDC42 ERBIN HSPA9 TXN MAPK8IP3 RILPL2 PAN3 SRP68 HGS GSK3A AUP1 PEX16 AKT1 TSC1 PTPN1 VAMP2 ACAA1 RPL34 RPL36 EIF4A3 FIP1L1 RPS3A DDX19B ACOX1 RPL9 RPS26 RPL23A

4.05E-09	119	Positive regulation of metabolic process	CD44 SBNO2 KAT6A MAP3K1 RBM23 RBM3 MED26 CHCHD2 CAMTA2 VDR DPF2 MAPK8IP3 ASXL2 LAMTOR1 ING1 ZNF296 CAMTA1 MAP3K5 APOPT1 TXN CD74 BAK1 SPI1 PICALM MAVS FUS MUL1 PYCARD LYL1 CDKN1B STAT1 ATF6 JCHAIN NOTCH2 UBQLN1 CDK5RAP2 PELI2 AKT1 HSPD1 OGT NOTCH1 YTHDF1 FLI1 KLF10 BTG2 ZC3H12A BRD7 LGALS9 ATF7IP CEBPB ZNF24 MLXIP AURKAIP1 TBL1XR1 ST20 CREB3L2 MAFF SP1 YTHDF3 BCAP31 SEMA4D PTPN1 XRCC6 GPATCH3 TMSB4X PDCD6 HIPK2 TAF11 TNFRSF1A MNT CDC42 EPS15 MAPK3 CSK NFKBIB USF2 BUD31 RAPGEF1 PDLIM1 MAK KCTD20 RAP1A MAPK8IP1 RAP2C CHI3L1 RANBP2 NPTN ADIPOR1 TSC1 CCS CHP1 DDX39B AIF1 DDT GSK3A C9orf72 RAB7A PPP2CA CCT4 RPS6KA1 CLN6 FBH1 HMGA1 EIF4A3 CACUL1 STX5 PIP4K2C RNF187 IRF2 SIN3A RNF139 HGS LILRA5 SIRT7 PRKAR1A SF3B1 TAB3 FPR1 HMGN1
4.05E-09	82	Protein transport	NPEPPS RAB7A SAR1A RAB2A SEC61B SNX17 TMED5 SPCS3 NAPG STX11 HSPD1 RAB5A GOLGA7 RABGAP1L RANBP2 ZDHHC7 STX5 ZDHHC3 CD14 TBC1D10C POM121 CHMP1B MAVS PYCARD CSK RAP1A ZC3H12A LGALS9 IFIT1 BCAP31 LILRA5 CHP1 DDX39B AIF1 TMSB4X PDCD6 LAMP2 CD74 GABARAPL2 FAM160A2 CYB5R4 CDC42 EPS15 MAPK3 IFT20 ERBIN HSPA9 CHI3L1 TXN MAPK8IP3 DOP1B RILPL2 PAN3 VPS8 DDX19B SRP68 HGS TAP2 NOTCH1 GSK3A AUP1 PEX16 PPT1 AKT1 TSC1 ARHGAP1 PTPN1 HCAR2 VAMP2 ACAA1 RPL34 CANX RPL36 ATP6V1G1 EIF4A3 FIP1L1 RPS3A ACOX1 RPL9 ATP6V0C RPS26 RPL23A
4.05E-09	49	Viral process	USF2 CD74 TAF11 MAVS TRIM25 ZC3H12A LGALS9 SP1 IFIT1 MCTS1 CHMP1B SPEN TNFRSF1A EPS15 MAPK3 SELPLG RNGTT SKP1 STAT1 HSPD1 DYNC1LI1 OGT F11R PSMB4 ATF7IP BCAP31 ATP6V0C ISG15 MAP3K5 PSMB8 TAP2 CDC42 RAB7A TOP2B RAB5A NOTCH1 ISG20 HIPK2 RPL34 RPL36 HMGA1 RPS3A RANBP2 ELMO1 RPL9 POM121 XRCC6 RPS26 RPL23A
4.05E-09	51	Symbiont process	USF2 CD74 TAF11 MAVS TRIM25 ZC3H12A LGALS9 SP1 IFIT1 DDX39B MCTS1 CHMP1B SPEN TNFRSF1A EPS15 MAPK3 SELPLG RNGTT SKP1 STAT1 HSPD1 DYNC1LI1 OGT F11R PSMB4 ATF7IP BCAP31 ATP6V0C ISG15 MAP3K5 PSMB8 TAP2 CDC42 RAB7A TOP2B RAB5A NOTCH1 ISG20 HIPK2 RPL34 CBL RPL36 HMGA1 RPS3A RANBP2 ELMO1 RPL9 POM121 XRCC6 RPS26 RPL23A
4.05E-09	104	Cellular localization	RAP1A CCT4 NPEPPS PICALM RAB7A SAR1A RAB2A SEC61B IFT20 MAK SNX17 TMED5 SPCS3 NAPG BLOC1S1 STX11 KIAA1109 RBM26 RHOT2 DOP1B HSPD1 RAB5A GOLGA7 LAMTOR1 RABGAP1L RANBP2 ZDHHC7 STX5 ZDHHC3 TBC1D10C FES BCAP31 POM121 TMSB4X CHMP1B EPS15 MAVS CSK PEX16 ATXN1 ZC3H12A RNF139 IFIT1 CHP1 DDX39B PDCD6 LAMP2 CD74 BAK1 CDC42 CBL ERBIN HSPA9 DBN1 ITGA4 CANX PPT1 TXN CDK5RAP2 ADAM10 MAPK8IP3 AKT1 RILPL2 PAN3 NPTN SRP68 SIN3A SLC30A1 CREB3L2 ACTG1 HGS NSFL1C UBXN2B FAM160A2 TNFRSF1A MUL1 GSK3A AUP1 OSBPL2 F11R DPY30 TSC1 LGALS9 ARHGAP1 PTPN1 TAP2 SKP1 VAMP2 ACAA1 CAPZB MAPK3 RPL34 PPP6C RPL36 HMGA1 EIF4A3 DYNC1LI1 FIP1L1 RPS3A DDX19B ACOX1 RPL9 RPS26 RPL23A
4.39E-09	112	Positive regulation of cellular metabolic	CD44 SBNO2 KAT6A MAP3K1 RBM23 RBM3 CHCHD2 CAMTA2 VDR DPF2 MAPK8IP3 ASXL2 LAMTOR1 ING1 ZNF296 CAMTA1 MAP3K5 APOPT1 TXN CD74 BAK1 SPI1 PICALM MAVS FUS MUL1 PYCARD LYL1 STAT1 ATF6 NOTCH2 UBQLN1 CDK5RAP2 PELI2 AKT1 HSPD1

		process	OGT NOTCH1 YTHDF1 FLI1 KLF10 BTG2 ZC3H12A BRD7 ATF7IP CEBPB ZNF24 MLXIP AURKAIP1 TBL1XR1 ST20 CREB3L2 MAFF SP1 YTHDF3 BCAP31 SEMA4D PTPN1 XRCC6 GPATCH3 TMSB4X PDCD6 HIPK2 TAF11 TNFRSF1A MNT CDC42 EPS15 MAPK3 CSK NFKBIB MED26 USF2 BUD31 RAPGEF1 PDLIM1 CDKN1B MAK KCTD20 RAP1A MAPK8IP1 RAP2C CHI3L1 RANBP2 NPTN TSC1 CHP1 DDX39B AIF1 DDT GSK3A C9orf72 PPP2CA CCT4 RPS6KA1 CLN6 FBH1 HMGA1 EIF4A3 CACUL1 PIP4K2C RNF187 IRF2 LGALS9 SIN3A RNF139 LILRA5 SIRT7 PRKAR1A TAB3 FPR1 HMGN1
7.76E-09	82	Peptide transport	NPEPPS RAB7A SAR1A RAB2A SEC61B SNX17 TMED5 SPCS3 NAPG STX11 HSPD1 RAB5A GOLGA7 RABGAP1L RANBP2 ZDHHC7 STX5 ZDHHC3 CD14 TBC1D10C POM121 TAP2 CHMP1B CD74 MAVS PYCARD CSK RAP1A ZC3H12A LGALS9 IFIT1 BCAP31 LILRA5 CHP1 DDX39B AIF1 TMSB4X PDCD6 LAMP2 GABARAPL2 FAM160A2 CYB5R4 CDC42 EPS15 MAPK3 IFT20 ERBIN HSPA9 CHI3L1 TXN MAPK8IP3 DOP1B RILPL2 PAN3 VPS8 DDX19B SRP68 HGS NOTCH1 GSK3A AUP1 PEX16 PPT1 AKT1 TSC1 ARHGAP1 PTPN1 HCAR2 VAMP2 ACAA1 RPL34 CANX RPL36 ATP6V1G1 EIF4A3 FIP1L1 RPS3A ACOX1 RPL9 ATP6V0C RPS26 RPL23A
7.76E-09	84	Establishment of protein localization	NPEPPS RAB7A SAR1A RAB2A SEC61B SNX17 TMED5 SPCS3 NAPG STX11 HSPD1 RAB5A GOLGA7 RABGAP1L RANBP2 ZDHHC7 STX5 ZDHHC3 CD14 TBC1D10C POM121 CHMP1B MAVS PYCARD CSK RAP1A ZC3H12A LGALS9 IFIT1 BCAP31 LILRA5 CHP1 DDX39B AIF1 TMSB4X PDCD6 LAMP2 CD74 GABARAPL2 FAM160A2 CYB5R4 CDC42 EPS15 MAPK3 IFT20 ERBIN HSPA9 CHI3L1 TXN MAPK8IP3 DOP1B RILPL2 PAN3 VPS8 DDX19B SRP68 HGS TAP2 NOTCH1 GSK3A AUP1 CCT4 PEX16 PPT1 AKT1 TSC1 ARHGAP1 PTP4A3 PTPN1 HCAR2 VAMP2 ACAA1 RPL34 CANX RPL36 ATP6V1G1 EIF4A3 FIP1L1 RPS3A ACOX1 RPL9 ATP6V0C RPS26 RPL23A
8.16E-09	108	Positive regulation of nitrogen compound metabolic process	CD44 SBNO2 KAT6A MAP3K1 RBM23 RBM3 CHCHD2 CAMTA2 VDR DPF2 MAPK8IP3 ASXL2 LAMTOR1 ING1 ZNF296 CAMTA1 MAP3K5 APOPT1 TXN CD74 BAK1 SPI1 PICALM MAVS FUS MUL1 PYCARD LYL1 CDKN1B STAT1 ATF6 NOTCH2 UBQLN1 CDK5RAP2 PELI2 AKT1 HSPD1 OGT NOTCH1 YTHDF1 FLI1 KLF10 BTG2 ZC3H12A BRD7 ATF7IP CEBPB ZNF24 MLXIP AURKAIP1 TBL1XR1 ST20 CREB3L2 MAFF SP1 YTHDF3 BCAP31 SEMA4D PTPN1 XRCC6 GPATCH3 TMSB4X PDCD6 HIPK2 TAF11 TNFRSF1A MNT CDC42 MAPK3 CSK NFKBIB MED26 USF2 BUD31 RAPGEF1 PDLIM1 MAK RAP1A MAPK8IP1 RAP2C CHI3L1 NPTN CHP1 DDX39B AIF1 DDT GSK3A RAB7A PPP2CA CCT4 RPS6KA1 CLN6 FBH1 HMGA1 EIF4A3 CACUL1 STX5 RNF187 IRF2 LGALS9 SIN3A RNF139 LILRA5 SIRT7 PRKAR1A TAB3 FPR1 HMGN1
1.29E-08	102	Intracellular signal transduction	MAVS MUL1 LGALS9 CD44 BAK1 MAPK6 CDC42 RAB7A MAP3K1 MAPK3 PYCARD RAB2A PRKAR1A MAK MAPK8IP3 RHOT2 AKT1 RAB5A LAMTOR1 TSC1 CD14 TNFAIP8L1 GNG2 MAP3K5 ARHGAP19 APOPT1 TXN CD74 WWC3 HIPK2 STAT1 TRIM25 NOTCH2 PELI2 RIT1 OGT NOTCH1 ZC3H12A BAG5 ARHGAP1 ATP6AP2 PTP4A3 HGS CHP1 PDCD6 TNFRSF1A POLB CSK RHEB RAPGEF1 CBL ERBIN RAP1A RPS6KA1 MAPK8IP1 RAP2C PSD4 CHI3L1 C9orf72 PIK3AP1 NPTN CDC42SE2 ADIPOR1 DUSP18 CEBPB PTPN1 GPATCH3 AIF1 DDT ELMO1 TMSB4X PPIF FBH1 UBQLN1 TAB3 CAMTA1 BCAP31 LILRA5 SEMA4D CEACAM1 GSK3A PPP2CA G3BP2 CARD19 TAF11 KAT6A CORO2A CDKN1B CNOT2 SKP1 ARAP3 LY96 PSMD4 PSMB4 BTG2 TOPBP1 BRD7 TAF10 PIP4K2C FPR1 PSMD13 PSMB8

1.07E-14	172	Response to organic substance	FOXRED2 TSC2 ZNF106 IL10RA MAPKAPK3 SERP1 MED1 ANXA1 ABHD2 NLRC5 IFITM3 IFNAR1 MARCH6 MAPKAPK2 TGFBR2 RAB8A SGTB UPF1 RUNX3 HERPUD1 ATP2B4 CYLD REST ICAM1 SNW1 PPM1A CARD8 PADI2 FFAR2 SNX6 DSTYK MDM2 TLR2 PAK1 SPATA2 ABCG1 CTNNB1 UBE2O MX2 CIB1 SELL DAXX ATF6B STRAP DGAT2 DNM2 ATRX SNX10 RFX2 GRAMD1A RANGAP1 YY1 TRIB3 EHD4 GAB1 KLF3 CHKA ARPC3 PDCD10 GNAI2 EIF4G1 STXBP3 RAD23B DNMT3A IFIT2 PTK2B HERPUD2 ITPR2 MBD4 LDLR KRAS TOR1A ACTR2 DUSP6 TOB1 ARHGDIA MTF2 ZEB1 EIF4EBP2 VPS26B CDK19 KAT6B EIF4A2 ZBTB7B RBM15 MNDA IFI16 NFIL3 INPPL1 TET2 PTAFR SYAP1 STX8 AKAP13 PRPF8 RPS6KA3 TAF7 PDE4B IRF7 STRN3 MYO5A NUB1 ST3GAL6 PSEN1 RTF2 ZFAND6 EZR RPS6KA5 RIOK3 EMD ZNF451 KLF7 PTPN12 CDKN1C LILRB2 LAMTOR5 KAT7 FLOT1 TRIM41 EPG5 UBXN1 H2AFZ KMT2D STAT2 BCL9L CD47 GET4 LILRA2 ACAP2 TCF7L2 GNAQ PIAS1 SHOC2 GAB2 PSMA4 ATP6V1H UBE2D1 ACADVL ADD1 DNAJB11 PSMD8 HNRNPM ATP6V1D PSME2 ARFGAP1 HCK XIAP POLR2C TYK2 PSMD3 UBE2D3 OAS3 TRIM38 ATP6V1A SUMO1 TNFSF14 RBCK1 PSME3 TLN1 MBTPS1 ARF1 HNRNPDL MX1 TNFRSF14 ATP6V0D1 UBB TALDO1 SRPRA HLA-DRA DCTN1 PSMB9 PSMB3
1.07E-14	151	Cellular response to organic substance	TSC2 ZNF106 IL10RA SERP1 MED1 ANXA1 ABHD2 NLRC5 IFITM3 IFNAR1 MAPKAPK2 TGFBR2 RAB8A UPF1 ATP2B4 CYLD REST ICAM1 SNW1 PPM1A CARD8 PADI2 FFAR2 SNX6 DSTYK MDM2 TLR2 PAK1 SPATA2 CTNNB1 UBE2O CIB1 DAXX ATF6B STRAP HERPUD1 DGAT2 DNM2 SNX10 RFX2 GRAMD1A RANGAP1 YY1 TRIB3 EHD4 KLF3 ARPC3 PDCD10 GNAI2 STXBP3 RAD23B DNMT3A IFIT2 PTK2B ITPR2 LDLR KRAS TOR1A ACTR2 TOB1 ARHGDIA MTF2 ZEB1 EIF4EBP2 VPS26B CDK19 KAT6B EIF4A2 ZBTB7B RBM15 MNDA IFI16 NFIL3 PTAFR SYAP1 STX8 AKAP13 PRPF8 TAF7 PDE4B IRF7 STRN3 MYO5A ST3GAL6 PSEN1 ZFAND6 EZR RPS6KA5 RIOK3 EMD GAB1 ZNF451 KLF7 PTPN12 CDKN1C LILRB2 LAMTOR5 FLOT1 TRIM41 EPG5 H2AFZ KMT2D STAT2 BCL9L CD47 LILRA2 ACAP2 GNAQ PIAS1 SHOC2 GAB2 PSMA4 ATP6V1H UBE2D1 ACADVL ADD1 DNAJB11 PSMD8 HNRNPM ATP6V1D PSME2 ARFGAP1 HCK XIAP POLR2C TYK2 PSMD3 UBE2D3 OAS3 TRIM38 ATP6V1A SUMO1 TNFSF14 RBCK1 PSME3 TLN1 MBTPS1 ARF1 HNRNPDL MX1 TNFRSF14 ATP6V0D1 INPPL1 UBB TALDO1 SRPRA MX2 HLA-DRA DCTN1 PSMB9 PSMB3
5.39E-13	166	Cellular response to chemical stimulus	TSC2 ZNF106 IL10RA SERP1 MED1 ANXA1 ABHD2 NLRC5 IFITM3 IFNAR1 MAPKAPK2 TGFBR2 RAB8A UPF1 ATP2B4 CYLD REST ADD1 ICAM1 SNW1 PPM1A CARD8 SUMO1 PADI2 FFAR2 SNX6 DSTYK MDM2 TLR2 PAK1 SPATA2 PINK1 S100A12 CTNNB1 UBE2O CIB1 DAXX ATF6B STRAP HERPUD1 DGAT2 DNM2 ATRX SNX10 RFX2 GRAMD1A MCOLN1 RANGAP1 YY1 TRIB3 EHD4 UBE2D3 KLF3 ARPC3 PDCD10 GNAI2 STXBP3 RAD23B DNMT3A IFIT2 KDM3B PTK2B ITPR2 TNFSF14 LDLR KDM6B KRAS TOR1A SLC31A1 ACTR2 TOB1 ARHGDIA MTF2 ZEB1 EIF4EBP2 VPS26B CDK19 KAT6B EIF4A2 ZBTB7B RBM15 MNDA IFI16 NFIL3 PTAFR SYAP1 STX8 AKAP13 PRPF8 ZFAND2A TAF7 CAMK1D PDE4B IRF7 STRN3 MYO5A ST3GAL6 PSEN1 BNIP3L RTF2 DAPK2 ZFAND6 EZR RPS6KA5 RIOK3 EMD GAB1 ZNF451 ATP6V1A KMT2A KLF7 CHD6 PTPN12 CDKN1C LILRB2 LAMTOR5 FLOT1 TRIM41 EPG5 ATP6V0D1 H2AFZ KMT2D STAT2 BCL9L CD47 LILRA2 ACAP2 GNAQ PIAS1 SHOC2 GAB2 PSMA4 ATP6V1H UBE2D1 ACADVL DNAJB11 PSMD8 HNRNPM ATP6V1D PSME2 ARFGAP1 HCK XIAP POLR2C TYK2 PSMD3 OAS3

			TRIM38 RBCK1 PSME3 EGLN1 TLN1 MBTPS1 ARF1 HNRNPDL MX1 TNFRSF14 INPPL1 UBB TALDO1 SRPRA MX2 HLA-DRA DCTN1 PSMB9 PSMB3
2.33E-12	192	Cellular protein modification process	SPAG9 UBE3C RB1CC1 RNF19A USP36 ST3GAL6 SLK UBE2D1 ITCH CYLD USP48 RPS6KA5 STK4 TRIB3 HCK TNKS2 SHOC2 CCNY UNC119 UBE2D3 MAPKAPK3 MOGS USP34 AAK1 SUMO1 PADI2 KDM3B SERP1 PTK2B RBCK1 HAT1 MGAT1 NAA35 KAT7 DUSP6 ESCO1 PIK3R5 MTF2 PAK1 TRIP12 MPPE1 CDK19 UBE2L6 KAT6B TNFRSF14 PINK1 MAPKAPK2 RNF181 UBB QARS UBE2F PPP1R2 PIGN MIER1 PJA2 CTNNB1 CDC27 BAZ1B RUNX3 PIAS1 DAPK2 ATP2B4 REST B4GALT1 SRPK1 PPM1A RNF125 XIAP SLC9A3R1 FBXO9 ZNF451 PDCD10 KMT2A FBXL5 MED1 TGM3 PTPN12 ANXA1 MDM2 EGLN1 TLR2 PPIG WARS MARCH6 TLK2 PTPRJ SMG1 DYRK1A SPATA2 FBXW5 VPS28 UBXN1 TGFBR2 ATXN7 ANAPC16 TET2 ALK UBE2O TAF7 PRKAG1 ZFP91 PHF2 GRK5 CHML DAXX ST3GAL1 HERPUD1 PSEN1 ICAM1 PPP2R3C RIOK3 MGRN1 CTCF EHD4 TYK2 KMT5B FBXO3 TRIM38 GNAI2 CNPPD1 SET CDKN1C KDM6B DSTYK KRAS FKBP11 AGAP2 ARRDC4 TRIM41 TRIM11 ZBTB7B SPSB3 MTMR14 PIGX RNF169 FBXO22 KMT2D SETD5 PXK ZEB2 SYAP1 AKAP13 PPP1CA RPS6KA3 GAK CAMK1D CIB1 STK40 GRK6 MAN1A2 PHACTR4 LCP2 NUB1 STRAP ATRX EZR SNW1 H2AFY EIF4G1 TOR1A FLOT1 JTB GNAQ CTBP1 CAMLG CEP295 STAT2 TSC2 LILRB2 EMC10 SNX6 PSMA4 SEH1L NUP50 PSMD8 APOL1 RANGAP1 YY1 PSME2 FBXL20 PSMD3 RAD23B PSME3 DBNL MBTPS1 S100A12 MBD6 RAB8A SERPINA1 PSMB9 PSMB3
2.88E-12	68	Regulation of cellular catabolic process	USP36 ABHD5 RNF19A SMG6 HNRNPM PSME2 TRIB3 PSME3 UPF1 HERPUD1 ATP2B4 IL10RA SUMO1 RAD23B PTK2B MDM2 TOB1 UBXN1 MAPKAPK2 UBB DCP2 GIGYF2 PIAS1 PSEN1 BNIP3L FBXL5 LDLR PINK1 FBXO22 PPP1CA ZFAND2A IFI16 EZR TSC2 NUB1 ITCH EIF4G2 EIF4G1 TENT4B VPS13C TLK2 CAMLG RAB8A PAFAH1B2 SUPT5H ATP6V1H ATP6V1D ATP6V1A SNX6 SERBP1 ATP6V0D1 RB1CC1 DAPK2 PSMA4 DIS3 SEH1L NUP50 PSMD8 PSMD3 EXOC8 SET LAMTOR5 VPS26B PFKFB3 PRKAG1 PSMB9 PSMB3
3.53E-12	198	Macromolecule modification	SPAG9 UBE3C RB1CC1 RNF19A USP36 ST3GAL6 SLK BUD23 UBE2D1 ITCH CYLD USP48 RPS6KA5 STK4 TRIB3 HCK TNKS2 SHOC2 CCNY UNC119 UBE2D3 MAPKAPK3 MOGS USP34 AAK1 SUMO1 PADI2 KDM3B SERP1 PTK2B RBCK1 HAT1 MGAT1 NAA35 KAT7 DUSP6 ESCO1 PIK3R5 MTF2 PAK1 TRIP12 MPPE1 CDK19 UBE2L6 KAT6B TNFRSF14 PINK1 MAPKAPK2 TET2 RNF181 UBB QARS UBE2F PPP1R2 PIGN MIER1 PJA2 CTNNB1 CDC27 BAZ1B RUNX3 PIAS1 DAPK2 ATP2B4 REST B4GALT1 SRPK1 PPM1A RNF125 XIAP SLC9A3R1 FBXO9 ZNF451 PDCD10 KMT2A FBXL5 DNMT3A MED1 TGM3 PTPN12 ANXA1 MDM2 EGLN1 TLR2 PPIG WARS MARCH6 TLK2 PTPRJ SMG1 DYRK1A SPATA2 FBXW5 VPS28 UBXN1 RBM15 TGFBR2 ATXN7 ANAPC16 ALK UBE2O TAF7 PRKAG1 ZFP91 PHF2 GRK5 CHML DAXX ST3GAL1 HERPUD1 PSEN1 ICAM1 PPP2R3C RIOK3 MGRN1 CTCF EHD4 TYK2 KMT5B FBXO3 TRIM38 GNAI2 CNPPD1 SET CDKN1C KDM6B DSTYK KRAS FKBP11 AGAP2 ARRDC4 TRIM41 TRIM11 ZBTB7B SPSB3 MTMR14 PIGX RNF169 FBXO22 KMT2D SETD5 PXK ZEB2 SYAP1 AKAP13 PPP1CA RPS6KA3 GAK CAMK1D CIB1 STK40 GRK6 MAN1A2 PHACTR4 LCP2 NUB1 STRAP ATRX EZR SNW1 H2AFY EIF4G1 TOR1A FLOT1 JTB GNAQ CTBP1 CAMLG CEP295 STAT2 TSC2 LILRB2 EMC10 SNX6 TSR3 PSMA4 SEH1L NUP50 PSMD8 APOL1 RANGAP1 YY1 PSME2 FBXL20

			PSMD3 RAD23B MBD4 PSME3 DBNL MBTPS1 S100A12 MBD6 RAB8A GATAD2A SERPINA1 PSMB9 PSMB3
4.53E-12	180	Organelle organization	SMARCD2 USP36 UBE2D3 PINK1 RB1CC1 ATG2B SMG6 NDE1 PCM1 DNM2 PDS5B ADD1 PPP2R3C SNAP23 ATP6V1D STAG2 BNIP3L TNKS2 CEP164 ARPC3 H2AFY GORASP2 WIPF1 PADI2 KDM3B SEPT7 HIP1 HAT1 CAP1 ANXA1 WASHC4 KAT7 MAP1LC3B ESCO1 MTF2 ARFGAP2 VPS51 ASAP1 TRIP12 KAT6B ATAD2 MX1 ARPC5 CDC42EP3 H2AFZ BRWD3 CEP295 RAB8A ATXN2L LMAN2 TAPT1 STX8 MX2 TUBB4B MCMBP MIER1 CAPZA2 ATG9A GET4 LILRB2 CTNNB1 UPF1 BAZ1B REST ATRX EZR RPS6KA5 HCK CTCF MTPN SLC25A36 KMT2A RAD23B KATNBL1 ACTR2 SERBP1 TLK2 PAK1 ATXN7 TAF7 CIB1 BLOC1S2 DAXX DCTN1 FARP2 BUD23 SMC1A PSEN1 CYLD ZFAND6 RFX2 ICAM1 YY1 SLC9A3R1 SLAIN2 KMT5B SUMO1 SET DNMT3A PTK2B CHD6 CDKN1C KDM6B KRAS CEP350 TMEM127 DBNL TOR1A TLN1 ARF1 DYRK1A FBXW5 ZBTB7B KMT2D SETD5 TET2 ZEB2 BRD3 UBB AKAP13 AGFG1 IKZF1 PHF2 MSRB1 PHACTR4 ODF2 CDC27 HEBP2 SLK AP1M1 SEH1L SNX10 SNW1 GAB1 ZNF451 SUB1 PDCD10 EIF4G1 EXOC8 TENT4B ZBTB1 VPS13C CHMP2A CLIP1 MBTPS1 ARHGDIA STRIP1 POGZ SH3KBP1 TCF7L2 SMG1 CTBP1 ARHGAP12 INPPL1 PDZD8 STAT2 DCP2 GAK ARAP1 PRPF40A FGD4 ATP6V0D1 CD47 TSC2 RNF19A ATP6V1H UBE2D1 SRPK1 CTSZ EMD ATP6V1A IFIT2 NFE2 ATP5F1E ALDOA VPS28 TRAPPC1 TRAPPC5 SERPINA1 DYNLL2
8.18E-12	72	Regulation of catabolic process	USP36 ABHD5 RNF19A SMG6 ITCH HNRNPM PSME2 TRIB3 PSME3 UPF1 HERPUD1 ATP2B4 IL10RA SUMO1 RAD23B PTK2B AGAP2 MDM2 TOB1 UBXN1 MAPKAPK2 UBB DCP2 GIGYF2 PIAS1 PSEN1 BNIP3L FBXL20 PSMD3 FBXL5 LDLR EGLN1 PINK1 FBXO22 PPP1CA ZFAND2A IFI16 EZR TSC2 NUB1 EIF4G2 EIF4G1 TENT4B VPS13C TLK2 VPS28 CAMLG RAB8A PAFAH1B2 SUPT5H ATP6V1H ATP6V1D ATP6V1A SNX6 SERBP1 ATP6V0D1 RB1CC1 DAPK2 PSMA4 DIS3 SEH1L NUP50 PSMD8 EXOC8 SET LAMTOR5 VPS26B PFKFB3 PRKAG1 PSMB9 PSMB3
2.82E-11	125	Cellular catabolic process	USP36 UPF1 UBE3C ABHD5 RB1CC1 RNF19A PSMA4 ATG2B SMG6 UBE2D1 ITCH DIS3 PSMD8 HNRNPM FOXRED2 PSME2 CTSZ TRIB3 GDPD3 FBXL20 PSMD3 UBE2D3 FBXO9 FBXL5 RAD23B TENT4B RBCK1 PSME3 ABHD2 MAP1LC3B MARCH6 EPG5 TRIP12 UBE2L6 SMG1 PINK1 VPS28 SPSB3 UBAP1 TET2 UBB DCP2 SGTB ATG9A PSMB9 RBM8A PSMB3 HERPUD1 ATP2B4 ACADVL XRN2 RNF125 IL10RA SUMO1 PTK2B MDM2 TOB1 FBXW5 UBXN1 MAPKAPK2 CTNNB1 GIGYF2 ABHD16A RNPS1 PIAS1 PSEN1 CYLD MCOLN1 USP48 BNIP3L PDE4C TRIM38 USP34 NAGK LDLR MGAT1 TOR1A ALDOA IFI16 FBXO22 RAB8A RAB24 PPP1CA ZFAND2A PDE4B DDAH2 PLEKHM1 EZR TSC2 NUB1 EIF4G2 EIF4G1 VPS13C TLK2 VPS51 CAMLG PAFAH1B2 SUPT5H GET4 ATP6V1H ATP6V1D ATP6V1A SNX6 SERBP1 ATP6V0D1 CDC27 DAPK2 SEH1L NUP50 RPL19 EXOC8 SET MBD4 CHMP2A LAMTOR5 VPS26B MTMR14 PGM2L1 ANAPC16 PFKFB3 PRKAG1 RPL41 DYNLL2 RPL17
7.44E-11	114	Vesicle-mediated transport	SPAG9 DNM2 SNAP23 ARFGAP1 UNC119 WIPF1 EXOC8 HIP1 CHMP2A ARF3 ANXA1 WASHC4 ARF1 ARFGAP2 VPS51 VPS26B RER1 VPS28 RAB8A LMAN2 TRAPPC1 STX8 GAK TRAPPC5 BLOC1S2 CD47 AP5B1 CUX1 ATP6V1H IL10RA AAK1 GDI2 AP1M1 PSEN1

			SNX10 LAT2 EZR UNC13D HCK EHD4 FBXL20 GNAI2 MAPKAPK3 STXBP3 LDLR CAP1 NUMB DBNL TOR1A FLOT1 SH3KBP1 PAK1 EPG5 MPPE1 MAPKAPK2 TGFBR2 CTNNB1 PTAFR MYO5A SYNRG MGRN1 SNX6 PINK1 ARHGAP12 INPPL1 UBE2O CAMK1D LAMP1 DCTN1 GAB2 REST TSC2 ACAP2 TLR2 PTPRJ DNAJC5 FCGRT CD93 SERPINB1 HEBP2 B4GALT1 APOL1 ATP6V1D CTSZ PSMD3 CYTH1 ARPC3 SCAMP3 PADI2 CTSD NPC2 FGL2 VPS13C LILRB2 TMBIM1 TLN1 ACTR2 SLC15A4 SERPING1 ALDOA ARPC5 S100A12 MNDA UBAP1 CLEC4D PAFAH1B2 RAB24 UBB DGAT1 TUBB4B SELL SERPINA1 CAPZA2 DYNLL2
1.26E-10	139	Negative regulation of macromolecule metabolic process	UPF1 SERPINB1 SMG6 HNRNPM XIAP TENT4B KAT7 DUSP6 NFIC EIF4EBP2 SERPING1 TRIP12 KAT6B ATAD2 SMG1 GATAD2A QARS DCP2 SERPINA1 MIER1 CUX1 RBM8A DNMT3A CYLD REST SNW1 RPS6KA5 YY1 TRIB3 NKAP CTCF CARD8 SLC9A3R1 EIF4G1 SUMO1 SET ZBTB1 SNX6 EAPP CDKN1C LAMTOR5 AGAP2 MDM2 WARS TOB1 ZEB1 TCF7L2 PTPRJ EIF4A2 DIP2A VPS28 UBXN1 MAPKAPK2 IFI16 ZNF148 RNF169 CTNNB1 ZEB2 CNBP RPS6KA3 TAF7 SUPT5H GIGYF2 DAXX RNPS1 STRAP PIAS1 HERPUD1 ITCH PSEN1 ATRX KLF3 H2AFY GNAI2 GON4L KHDRBS1 TTF1 MED1 TNFSF14 HAT1 LDLR MTF2 TNFAIP8 TRIM11 DYRK1A PINK1 CTBP1 ZBTB7B NFIL3 INPPL1 PPP1R2 CIB1 STRN3 PHACTR4 SMC1A EZR CTSZ STAG2 TNKS2 ZNF451 PDCD10 KMT2A KLF7 TLK2 GNAQ CAMLG PHF2 RUNX3 SFSWAP TSC2 LILRB2 KMT2D IKZF1 NACA ATP2B4 SERBP1 PSMA4 UBE2D1 DIS3 SEH1L NUP50 PSMD8 PPM1A PSME2 POLR2C RPL19 PSMD3 UBE2D3 TSNAX PSME3 UBB IRF7 RPL41 HSBP1 PSMB9 RPL17 PSMB3
1.26E-10	104	Intracellular transport	USP36 UBE2D3 PINK1 NDE1 PCM1 DNM2 SNAP23 NUP50 AFTPH VPS13C CHMP2A ARF3 COX5B WASHC4 ARF1 ARFGAP2 VPS51 VPS26B VPS28 RAB8A LMAN2 RAB24 TRAPPC1 STX8 TRAPPC5 SRPRA BLOC1S2 SGTB AP5B1 ATP5F1E SPAG9 RANGAP1 NPC2 KHDRBS1 UBB AP1M1 PSEN1 ZFAND6 LAT2 PPM1A EHD4 BNIP3L FBXL20 MED1 SNX6 CLIP1 MDM2 TOR1A ACTR2 MBTPS1 PAK1 EPG5 MPPE1 ABCG1 FBXO22 CTNNB1 AKAP13 PRKAG1 MYO5A CHML DCTN1 EZR HERPUD1 SNX13 UNC13D EMD MGRN1 SLC9A3R1 OSBP PDCD10 LDLR TCF7L2 UBE2O GAK MX2 CIB1 LAMP1 GAB2 TSC2 ACAP2 UPF1 SMG6 UBE2D1 SEH1L DNAJC5 CTSZ ARFGAP1 RPL19 HIP1 SMG1 U2AF1 SSR2 PSIP1 UBAP1 CDC40 AGFG1 SERPINA1 CAPZA2 RNPS1 RPL41 DYNLL2 RBM8A RPL17 SYNRG
1.04E-09	144	Negative regulation of metabolic process	UPF1 SERPINB1 SMG6 HNRNPM XIAP TENT4B KAT7 DUSP6 NFIC EIF4EBP2 SERPING1 TRIP12 KAT6B ATAD2 SMG1 GATAD2A QARS DCP2 SERPINA1 MIER1 CUX1 RBM8A DNMT3A ATP2B4 CYLD REST SNW1 RPS6KA5 YY1 TRIB3 NKAP CTCF CARD8 SLC9A3R1 IL10RA EIF4G1 SUMO1 SET ZBTB1 SNX6 EAPP CDKN1C LAMTOR5 AGAP2 MDM2 WARS TOB1 ZEB1 TCF7L2 PTPRJ EIF4A2 DIP2A VPS28 UBXN1 MAPKAPK2 IFI16 ZNF148 RNF169 CTNNB1 ZEB2 CNBP RPS6KA3 TAF7 SUPT5H GIGYF2 DAXX RNPS1 STRAP PIAS1 HERPUD1 DGAT2 ACADVL ITCH PSEN1 ATRX KLF3 H2AFY GNAI2 GON4L KHDRBS1 TTF1 MED1 TNFSF14 HAT1 LDLR MTF2 TNFAIP8 TRIM11 DYRK1A PINK1 CTBP1 ZBTB7B NFIL3 INPPL1 PPP1R2 CIB1 STRN3 PHACTR4 USP36 SMC1A EZR CTSZ STAG2 TNKS2 EIF4G2 ZNF451 PDCD10 KMT2A KLF7 TLK2 GNAQ CAMLG PHF2 RUNX3 SFSWAP TSC2 LILRB2 KMT2D IKZF1 NACA SERBP1 PSMA4 UBE2D1 DIS3 SEH1L NUP50 PSMD8 PPM1A PSME2 POLR2C RPL19 PSMD3 UBE2D3 TSNAX

			PSME3 UBB IRF7 RPL41 HSBP1 PSMB9 RPL17 PSMB3
1.04E-09	154	Immune system process	RUNX3 PPP2R3C BNIP3L FCGRT BTN3A3 MAPKAPK3 IFIT2 ANKHD1 ANXA1 TLR2 NLRC5 IFITM3 MX1 TNFRSF14 MAPKAPK2 TET2 MX2 IRF7 SRPK1 LILRB2 LCP2 CYLD LAT2 APOL1 RNF125 OAS3 PADI2 TNFSF14 RBCK1 TAPBPL IFNAR1 SERPING1 PTPRJ RBM15 S100A12 TGFBR2 IFI16 CD47 HLA-DRA LILRA2 FARP2 DAPK2 ROGDI APBB1IP ITCH PSEN1 REST B4GALT1 SNX10 ADD1 ICAM1 MCOLN1 UNC13D STK4 HCK RIOK3 NKAP ZMIZ1 FBXO9 TRIM38 STXBP3 GON4L PTK2B MED1 FFAR2 ZBTB1 CDKN1C LDLR KDM6B DBNL ACTR2 SECTM1 TMEM91 ZEB1 VPS26B TRIM11 ZBTB7B MNDA NFIL3 INPPL1 CLEC4D CTNNB1 PTAFR STX8 STAT2 RPS6KA3 CYBC1 CAMK1D PDE4B CIB1 SELL MSRB1 PSMB9 NUB1 GAB2 SNAP23 EZR FLOT1 EPG5 IKZF1 LAMP1 PJA2 CD93 TUBB4B SERPINB1 PIAS1 PSMA4 HEBP2 GDI2 UBE2D1 AP1M1 DNM2 PSMD8 ATP6V1D RPS6KA5 PSME2 DNAJC5 CTSZ XIAP TYK2 PSMD3 UBE2D3 ELF2 ARPC3 HRH2 WIPF1 SUMO1 CTSD KMT2A NPC2 NFE2 S1PR4 FGL2 CAP1 PSME3 KRAS TMBIM1 SLC15A4 IGSF6 ARF1 PAK1 ALDOA ARPC5 KMT2D PAFAH1B2 RAB24 TRAPPC1 UBB DGAT1 SERPINA1 CAPZA2 DCTN1 DYNLL2 PSMB3
2.81E-07	120	Organelle organization	SMARCA4 SH3GLB1 RAC2 UBE2J2 BRCA1 KDM5A XRCC5 SUPT16H RAB18 MAST3 FBXO7 DOT1L NEDD9 PHF1 CCND3 PHF13 CAPZA1 KDM5B NRDE2 RPL5 SIN3B MACF1 TUBGCP2 HAUS8 NASP DYNC1LI2 GCC2 PARVG WHAMM UQCRB PADI4 ALKBH4 MAP1A TPM4 NCKAP5L CHAF1A ATG4B TADA3 SAMD9L PLEC RFLNB SPG7 FCHSD1 RPL12 ATXN2 XRN1 FMR1 KAT8 HPS1 UBE2S ASH1L TNFSF10 CHMP1A PPHLN1 SMARCA5 XPC USP16 H3F3A DTX3L ABCA1 CRK PRELID1 ASXL1 CTDNEP1 MTHFR SETD2 C6orf89 NAP1L4 PUM2 HMG20B NCOA1 AKAP8 CDC37 PHACTR1 RO60 CREB1 PANK2 MKLN1 PPFIA1 KMT5C DOCK2 AGTPBP1 ARRB1 GINS4 MYSM1 MID1IP1 NSD1 ING2 KIF5B PAX5 SIPA1L1 SMURF1 NELFE RAD1 CHMP4A AKAP8L PNKP ARFGEF1 MAP2K7 TFEB IL1B IFI6 TMEM165 CLTC SCLT1 RPGR SYNJ1 TAOK1 ZNF274 SEPT9 PPP1R10 CORO7 FGD3 BIN3 GABPB1 ATP6V1E1 MT-ND6 F5 CSNK1E
7.41E-07	31	Covalent chromatin modification	BRCA1 PHF1 SIN3B PADI4 TADA3 KDM5A FMR1 KAT8 DOT1L ASH1L KDM5B PPHLN1 USP16 DTX3L ASXL1 MTHFR SETD2 C6orf89 NCOA1 KMT5C ARRB1 MYSM1 NSD1 ING2 PAX5 NELFE AKAP8L AKAP8 ZNF274 IL1B
7.41E-07	30	Histone modification	BRCA1 PHF1 SIN3B PADI4 TADA3 KDM5A FMR1 KAT8 DOT1L ASH1L KDM5B USP16 DTX3L ASXL1 MTHFR SETD2 C6orf89 NCOA1 KMT5C ARRB1 MYSM1 NSD1 ING2 PAX5 NELFE AKAP8L AKAP8 ZNF274 IL1B
7.41E-07	104	Positive regulation of cellular metabolic process	DBF4 BRCA1 PSMC4 CYFIP2 TP53BP1 MAP2K7 NCOA1 SUPT16H FBXO7 FMR1 PGK1 KAT8 GABPB1 MED25 PHF1 IL1B CCNT1 IL1RN CASP1 IGF1R RBMX TAOK1 CDK12 MAP4K2 ING2 DHRSX ASXL1 CASP4 NCOA6 CSNK1E ZNF350 ID2 AKAP8L BIRC3 CYBA KDM5A ARAF NRDC XRCC5 RBM22 PRPF6 RPRD1B DOT1L UBE2S NFKB1 NEDD9 TFEB CCND3 CEBPZ MEF2D ZBTB17 CREB1 ELF1 TNFSF10 RPL5 SMARCA4 HELZ2 TNNI2 F12 PPHLN1 MED4 ARRB1 WDFY2 ETS2 NSD1 RBPJ PRELID1 TADA3 DDIT3 C6orf89 SH3GLB1 CRKL GTPBP1 CST3 VRK3 CDC37 NCL ASH1L PUM1 SMARCA5 USP16 MID1IP1 NFATC2IP ARID3B ZBTB20 PAX5 SMURF1 NELFE DTX3L

			CTDNEP1 PNKP ARFGEF1 AKAP8 VHL MYSM1 PARP14 PPP1R10 ABCA7 EDF1 PRKAR2A CXCR4 ADCY4 ADM CRK
7.51E-07	41	Chromatin organization	SMARCA4 BRCA1 KDM5A SUPT16H DOT1L PHF1 KDM5B NRDE2 SIN3B NASP PADI4 CHAF1A TADA3 FMR1 KAT8 ASH1L PPHLN1 SMARCA5 USP16 H3F3A DTX3L ASXL1 MTHFR SETD2 C6orf89 NAP1L4 HMG20B NCOA1 PHF13 KMT5C ARRB1 MYSM1 NSD1 ING2 PAX5 NELFE AKAP8L AKAP8 ZNF274 IL1B
1.03E-06	109	Positive regulation of metabolic process	DBF4 BRCA1 PSMC4 CYFIP2 TP53BP1 MAP2K7 NCOA1 SUPT16H FBXO7 FMR1 PGK1 KAT8 GABPB1 MED25 PHF1 IL1B CCNT1 IL1RN CASP1 IGF1R RBMX TAOK1 CDK12 MAP4K2 ING2 DHRSX ASXL1 CASP4 NCOA6 SMURF1 CSNK1E ZNF350 ID2 AKAP8L BIRC3 CYBA KDM5A ARAF NRDC XRCC5 RBM22 PRPF6 RPRD1B DOT1L UBE2S NFKB1 NEDD9 TFEB CCND3 CEBPZ MEF2D ZBTB17 CREB1 ELF1 TNFSF10 RPL5 SMARCA4 HELZ2 TNNI2 F12 PPHLN1 MED4 ARRB1 WDFY2 ETS2 NSD1 RBPJ PRELID1 TADA3 DDIT3 C6orf89 EGLN2 MYLIP SH3GLB1 CRKL GTPBP1 CST3 VRK3 CDC37 NCL ASH1L KDM5B PUM1 SMARCA5 USP16 MID1IP1 ATG4B SLC50A1 NFATC2IP ARID3B ZBTB20 PAX5 NELFE DTX3L CTDNEP1 PNKP ARFGEF1 AKAP8 VHL MYSM1 PARP14 PPP1R10 ABCA7 CXCR4 EDF1 PRKAR2A ADCY4 ADM CRK
1.03E-06	92	Negative regulation of macromolec ule metabolic process	BRCA1 BIRC3 FMR1 KAT8 DOT1L NRDE2 IFI6 SIN3B HELZ2 RBPJ MAF1 CNOT10 ZNF350 NFKB1 XRN1 PUM2 TP53BP1 CST3 PRKAR2A ID2 KDM5B CREB1 RPL5 SRSF6 SMARCA4 CHMP1A VHL PPHLN1 PUM1 ARRB1 IGF1R CLTC HDGF SAP18 ETS2 DDIT3 CD55 AP2A1 CARD16 HMG20B KDM5A SRRT FBXO7 GTPBP1 PPP1R37 VRK3 EIF3A HIPK3 PHACTR1 CCND3 ASH1L PSPC1 SLPI IL1B NONO SMARCA5 ZNF274 ZBTB20 SIGIRR PAX5 NELFE PPP1R10 LRPAP1 PNKP XRCC5 IGBP1 CRKL MED25 PHF1 NCL GBP1 CCNT1 OS9 DTX3L PIP5KL1 USP47 PARP14 ATXN2 ABCA7 RBMX NSD1 MAP1A N4BP2L2 ZBED6 CLN8 PSMC4 PSMA7 H3F3A PSMD1 PSMD2 RPL12
1.03E-06	16	Regulation of histone modification	BRCA1 PHF1 FMR1 MTHFR C6orf89 KDM5A ING2 TADA3 PAX5 NELFE AKAP8L AKAP8 ARRB1 NSD1 ZNF274 IL1B
1.03E-06	86	Negative regulation of cellular metabolic process	BRCA1 BIRC3 FMR1 KAT8 DOT1L NRDE2 IFI6 SIN3B RBPJ MAF1 CNOT10 ZNF350 NFKB1 XRN1 TP53BP1 CST3 PPP1R37 PRKAR2A ID2 KDM5B CREB1 RPL5 SRSF6 MPHOSPH10 SMARCA4 CHMP1A VHL PPHLN1 ARRB1 IGF1R CLTC HDGF SAP18 ETS2 DDIT3 CD55 AP2A1 CARD16 HMG20B KDM5A FBXO7 HERC1 VRK3 EIF3A HIPK3 PHACTR1 CCND3 ASH1L PSPC1 SLPI IL1B PUM1 NONO SMARCA5 ZNF274 ZBTB20 SIGIRR PAX5 NELFE PPP1R10 LRPAP1 PNKP XRCC5 IGBP1 CRKL MED25 PHF1 NCL GBP1 CCNT1 OS9 DTX3L PIP5KL1 USP47 PARP14 ATXN2 CHMP4A ABCA7 RBMX NSD1 MAP1A N4BP2L2 ZBED6 CLN8 H3F3A
1.03E-06	87	Regulation of cellular protein metabolic process	BRCA1 BIRC3 CYFIP2 MAP2K7 FMR1 PHF1 CCND3 IL1B IFI6 IL1RN CASP1 IGF1R TAOK1 MAP4K2 CNOT10 CASP4 CSNK1E NFKB1 ARAF NRDC XRCC5 CST3 UBE2S NEDD9 PRKAR2A TNFSF10 RPL5 F12 ARRB1 WDFY2 PRELID1 MTHFR CD55 C6orf89 CARD16 DBF4 MYLIP PSMC4 PUM2 HMG20B KDM5A MKNK1 IGBP1 CRKL FBXO7 MTG2 PPP1R37 VRK3 CDC37 EIF3A HIPK3 PHACTR1 XRN1 ASH1L SLPI RAC2 CCNT1 PUM1 ING2 TADA3 PAX5 NELFE PPP1R10 AKAP8L PNKP ARFGEF1 PUM3 AKAP8 NCL GBP1 OS9 USP16 DTX3L NSD1 PIP5KL1 CDK12 USP47 ZNF274 PARP14 SMURF1 ABCA7 MAP1A

			CLN8 ATXN2 CXCR4 ADCY4 CRK
1.07E-06	92	Regulation of protein metabolic process	BRCA1 BIRC3 CYFIP2 MAP2K7 FMR1 PHF1 CCND3 IL1B IFI6 IL1RN CASP1 IGF1R TAOK1 MAP4K2 CNOT10 CASP4 SMURF1 CSNK1E NFKB1 ARAF NRDC XRCC5 CST3 UBE2S NEDD9 PRKAR2A TNFSF10 RPL5 F12 ARRB1 WDFY2 PRELID1 MTHFR CD55 C6orf89 CARD16 EGLN2 DBF4 MYLIP PSMC4 PUM2 HMG20B KDM5A MKNK1 IGBP1 CRKL FBXO7 MTG2 PPP1R37 VRK3 CDC37 EIF3A HIPK3 PHACTR1 XRN1 ASH1L SLPI RAC2 CCNT1 PUM1 ATG4B ING2 TADA3 PSMD1 PSMD2 SIGIRR PAX5 NELFE PPP1R10 AKAP8L PNKP ABCA7 ARFGEF1 PUM3 AKAP8 NCL GBP1 OS9 USP16 DTX3L NSD1 PIP5KL1 CDK12 USP47 ZNF274 PARP14 MAP1A CLN8 ATXN2 CXCR4 ADCY4 CRK
1.31E-06	99	Positive regulation of nitrogen compound metabolic process	DBF4 BRCA1 PSMC4 CYFIP2 TP53BP1 MAP2K7 NCOA1 SUPT16H FMR1 PGK1 KAT8 GABPB1 MED25 PHF1 IL1B CCNT1 IL1RN CASP1 IGF1R RBMX TAOK1 CDK12 MAP4K2 ING2 ASXL1 CASP4 NCOA6 SMURF1 CSNK1E ZNF350 ID2 AKAP8L BIRC3 KDM5A ARAF NRDC XRCC5 RBM22 PRPF6 RPRD1B DOT1L UBE2S NFKB1 NEDD9 TFEB CCND3 CEBPZ MEF2D ZBTB17 CREB1 ELF1 TNFSF10 RPL5 SMARCA4 HELZ2 TNNI2 F12 PPHLN1 MED4 ARRB1 WDFY2 ETS2 NSD1 RBPJ PRELID1 TADA3 DDIT3 C6orf89 EGLN2 MYLIP CRKL GTPBP1 CST3 VRK3 NCL ASH1L PUM1 SMARCA5 USP16 ATG4B NFATC2IP ARID3B ZBTB20 PAX5 NELFE DTX3L PNKP ARFGEF1 AKAP8 VHL MYSM1 PARP14 PPP1R10 ABCA7 EDF1 PRKAR2A CXCR4 ADCY4 CRK
1.51E-06	96	Negative regulation of metabolic process	BRCA1 BIRC3 FMR1 KAT8 DOT1L NRDE2 IFI6 SIN3B HELZ2 RBPJ MAF1 CNOT10 ZNF350 NFKB1 XRN1 PUM2 TP53BP1 CST3 PPP1R37 PRKAR2A ID2 KDM5B CREB1 RPL5 SRSF6 MPHOSPH10 IL1B SMARCA4 CHMP1A VHL PPHLN1 PUM1 ARRB1 IGF1R CLTC HDGF SAP18 ETS2 DDIT3 CD55 AP2A1 CARD16 HMG20B KDM5A SRRT FBXO7 GTPBP1 HERC1 VRK3 EIF3A HIPK3 PHACTR1 CCND3 ASH1L PSPC1 SLPI NONO SMARCA5 ZNF274 ZBTB20 SIGIRR PAX5 NELFE PPP1R10 LRPAP1 PNKP XRCC5 IGBP1 CRKL MED25 PHF1 NCL GBP1 CCNT1 OS9 DTX3L PIP5KL1 USP47 PARP14 ATXN2 CHMP4A ABCA7 NRDC RBMX NSD1 MAP1A N4BP2L2 ZBED6 CLN8 PSMC4 PSMA7 H3F3A PSMD1 PSMD2 RPL12
2.33E-06	81	Negative regulation of nitrogen compound metabolic process	BRCA1 BIRC3 FMR1 KAT8 DOT1L NRDE2 IFI6 SIN3B RBPJ MAF1 CNOT10 ZNF350 NFKB1 XRN1 TP53BP1 CST3 PRKAR2A ID2 KDM5B CREB1 RPL5 SRSF6 SMARCA4 CHMP1A VHL PPHLN1 ARRB1 IGF1R CLTC HDGF SAP18 ETS2 DDIT3 CD55 AP2A1 CARD16 HMG20B KDM5A FBXO7 PPP1R37 VRK3 EIF3A HIPK3 PHACTR1 CCND3 ASH1L PSPC1 SLPI IL1B PUM1 NONO SMARCA5 ZNF274 ZBTB20 SIGIRR PAX5 NELFE PPP1R10 PNKP XRCC5 IGBP1 CRKL MED25 PHF1 NCL GBP1 CCNT1 OS9 DTX3L PIP5KL1 USP47 PARP14 ABCA7 RBMX NSD1 MAP1A N4BP2L2 ZBED6 CLN8 H3F3A
3.47E-06	17	Regulation of chromatin organization	BRCA1 PHF1 FMR1 PPHLN1 MTHFR C6orf89 KDM5A ING2 TADA3 PAX5 NELFE AKAP8L AKAP8 ARRB1 NSD1 ZNF274 IL1B

Supplementary Table 6. Model training and validation samples and image information for **(a)** Circulating Cell Classifier and **(b)** Lung Tumor Classifier.

(a)

Circulating Cell Classifier Training Set		
**Cell Type**	**# Samples**	**# Images**
PBMCs	44	22,000,000
Fetal Blood Samples	18	2,800,000
NSCLC Cell Lines	4	156,000
HCC Cell Lines	4	400,000
Cells Lines (other)	4	440,000
**Total Training Sets**	**74**	**25,796,000**

Circulating Cell Classifier Validation Set		
**Cell Type**	**# Samples**	**# Images**
PBMCs	13	31,343,456
Fetal Blood Samples	37	10,174,532
NSCLC Cell Lines	1	632,086
HCC Cell Lines	1	162,906
Cells Lines (other)	3	725,492
**Total Validation Sets**	**55**	**43,038,472**

(b)

Lung Tumor Classifier Training Set		
**Cell Type**	**# Samples**	**# Images**
NSCLC	3	1,204,612
Lung Epithelial (normal)	2	1,678,862
Fibroblast	3	1,172,382
Endothelial	3	1,681,244
Smooth Muscle	3	1,555,220
M1 Macrophage	1	482,590
M2 Macrophage	1	414,766
Monocytes	2	1,536,930
Neutrophils	1	411,726
Natural Killer Cell	2	2,271,240
CD4 Activated T Cell	2	1,595,290
CD4 T Naive	3	2,176,754
CD4 T Central Memory	1	193,834
CD4 T Effector Memory	1	502,530
CD4 T Terminal Effector	1	466,096
CD8 T Naive	1	472,496
CD8 T Central Memory	1	476,432
CD8 T Effector Memory	1	291,462
CD8 T Terminal Effector	1	369,704
B Cell	2	1,682,748
Other immune (not listed above)	1	1,558,792
**Total Training Sets**	**36**	**22,195,710**

Lung Tumor Classifier Validation Set		
**Cell Type**	**# Samples**	**# Images**
NSCLC	2	1,371,576
Lung Epithelial (normal)	2	1,957,892
Fibroblast	2	1,763,040
Endothelial	2	1,504,912
Smooth Muscle	2	1,141,498
M1 Macrophage	1	418,850
M2 Macrophage	1	483,952
Monocytes	2	766,090
Neutrophils	1	437,728
Natural Killer Cell	2	1,220,308
CD4 Activated T Cell	2	1,643,760
CD4 T Naive	4	2,484,706
CD4 T Central Memory	1	88,108
CD4 T Effector Memory	1	423,664
CD4 T Terminal Effector	1	408,230
CD8 T Naive	1	466,632
CD8 T Central Memory	1	473,610
CD8 T Effector Memory	1	598,528
CD8 T Terminal Effector	1	475,764
B Cell	2	860,582
Other immune (not listed above)	1	1,693,228
**Total Validation Sets**	**33**	**20,682,658**

Supplementary Table 7. DTC imaging and sorting on COSMOS information

Run	Aliquot	COSMOS instrument	No. Cells imaged	Event rate (images/min)	Positive cells by classifier	Classifier Positive Rate	Run duration (hr)
1	Aliquot 1	1	62520	509.4	1132	1.81%	2.04
		2	73505	601.2	313	0.43%	2.04
	Aliquot 2	1	71507	1012.2	1000	1.40%	1.18
		2	63998	969	386	0.60%	1.10
2	Aliquot 3	1	27998	210.51	572	2.0%	2.13
		2	25194	208.21	557	2.2%	2.01
	Aliquot 4	1	25973	204.51	544	2.1%	2.07
		2	24995	210.04	635	2.5%	1.59

References

1. Suzuki, S. (1985). Topological structural analysis of digitized binary images by border following. Computer vision, graphics, and image processing, 30(1), 32-46.
