# Supplementary material for: COSMOS: a platform for real-time morphology-based, label-free cell sorting using deep learning
Source: Commun Biol. 2023 Sep 22;6:971. doi: 10.1038/s42003-023-05325-9 (PMC10516940; doi:10.1038/s42003-023-05325-9)
Supplement: Supplementary file 2 — Description of Additional Supplementary Files [file 42003_2023_5325_MOESM2_ESM.pdf]

Description of Additional Supplementary Files

File name: Supplementary Data 1

Description: The source data for Figure 3c-f: Processed whole genome sequencing and targeted mutation data from this study.
