# Supplementary material for: COSMOS: a platform for real-time morphology-based, label-free cell sorting using deep learning
Source: Commun Biol. 2023 Sep 22;6:971. doi: 10.1038/s42003-023-05325-9 (PMC10516940; doi:10.1038/s42003-023-05325-9)
Supplement: Supplementary file 4 — Reporting Summary [file 42003_2023_5325_MOESM4_ESM.pdf]

Corresponding author(s): Mahyar Salek

Last updated by author(s): Aug 28, 2023

Reporting Summary

Nature Portfolio wishes to improve the reproducibility of the work that we publish. This form provides structure for consistency and transparency in reporting. For further information on Nature Portfolio policies, see our Editorial Policies and the Editorial Policy Checklist.

Statistics

For all statistical analyses, confirm that the following items are present in the figure legend, table legend, main text, or Methods section.

n/a	Confirmed	
☐	☒	The exact sample size (*n*) for each experimental group/condition, given as a discrete number and unit of measurement
☐	☒	A statement on whether measurements were taken from distinct samples or whether the same sample was measured repeatedly
☐	☒	The statistical test(s) used AND whether they are one- or two-sided<br>*Only common tests should be described solely by name; describe more complex techniques in the Methods section.*
☒	☐	A description of all covariates tested
☒	☐	A description of any assumptions or corrections, such as tests of normality and adjustment for multiple comparisons
☐	☒	A full description of the statistical parameters including central tendency (e.g. means) or other basic estimates (e.g. regression coefficient) AND variation (e.g. standard deviation) or associated estimates of uncertainty (e.g. confidence intervals)
☐	☒	For null hypothesis testing, the test statistic (e.g. *F*, *t*, *r*) with confidence intervals, effect sizes, degrees of freedom and *P* value noted<br>*Give P values as exact values whenever suitable.*
☒	☐	For Bayesian analysis, information on the choice of priors and Markov chain Monte Carlo settings
☒	☐	For hierarchical and complex designs, identification of the appropriate level for tests and full reporting of outcomes
☐	☒	Estimates of effect sizes (e.g. Cohen's *d*, Pearson's *r*), indicating how they were calculated

Our web collection on statistics for biologists contains articles on many of the points above.

Software and code

Policy information about availability of computer code

Data collection	Custom software was developed for image annotation, which contains a command-line tool for clustering and annotating cell images with cell class labels. We also developed a custom deep learning model software for training and validating models, and is implemented using TensorFlow v1.15 and Nvidia TensorRT (v7.0.0).
Data analysis	Bioinformatics differential expression analysis was done using DESeq2 and iDEPCode. scRNA-Seq data was analyzed using the BDTM Data View v1.2.2 software (BD Biosciences, CA) software. Codes to reproduce all molecular analysis and figures are deposited to GitHub. Software code and data are available at https://github.com/deepcell/Salek_2022 and includes scripts used to generate ROC curves, confusion matrices, mutation allele counts, and CNV plots.

For manuscripts utilizing custom algorithms or software that are central to the research but not yet described in published literature, software must be made available to editors and reviewers. We strongly encourage code deposition in a community repository (e.g. GitHub). See the Nature Portfolio guidelines for submitting code & software for further information.

Data

Policy information about availability of data

All manuscripts must include a data availability statement. This statement should provide the following information, where applicable:

- Accession codes, unique identifiers, or web links for publicly available datasets
- A description of any restrictions on data availability
- For clinical datasets or third party data, please ensure that the statement adheres to our policy

Cell images used to generate presented UMAPs and associated embeddings, predictions, and labels are publicly available at https://github.com/deepcell/Salek_2022. Code to reproduce representative types of analysis and figures will be deposited to GitHub, see Supplementary Materials for more information. All processed molecular data (scRNA-Seq, whole genome sequencing, targeted mutation data) and corresponding scripts to generate figures are available on an S3 bucket and Github, respectively. Raw data can be provided upon reasonable request.

Human research participants

Policy information about studies involving human research participants and Sex and Gender in Research.

Reporting on sex and gender	No human research participants were included in this study.
Population characteristics	N/A
Recruitment	N/A
Ethics oversight	N/A

Note that full information on the approval of the study protocol must also be provided in the manuscript.

Field-specific reporting

Please select the one below that is the best fit for your research. If you are not sure, read the appropriate sections before making your selection.

☒ Life sciences ☐ Behavioural & social sciences ☐ Ecological, evolutionary & environmental sciences

For a reference copy of the document with all sections, see nature.com/documents/nr-reporting-summary-flat.pdf

Life sciences study design

All studies must disclose on these points even when the disclosure is negative.

Sample size	For training the CNN classifier, we used a data set of 25.7 million cells including 44 normal adult blood samples, 18 fetal blood samples, 4 NSCLC cell lines, 4 HCC cell lines and 4 other cell lines. A separate data set of 25.1 million cells from 111 samples of the cell types were used to validate the results f the classifier. The number of samples and images were chosen to try to represent a large enough set of different sample and cell types and to achieve high sensitivity and specificity performance of the classifier in distinguishing the different cell types. For NSCLC DTCs, 3 samples were chosen to represent samples with high, medium and low tumor cells frequencies.
Data exclusions	No exclusions.
Replication	For enrichment from cell spike-ins, 16 runs of PBMC spike-ins and 4 runs of whole blood spike-ins (including biological and technical replicates) were conducted to demonstrated enrichment of different cells from different initial frequencies and the compatibility with SNP and mutation analysis. For enrichment from NSCLC DTCs, 4 aliquots of the same samples were both run on two different instruments and showed similar enrichment ratios. For cell viability studies, replicates of same PBMC and cell line samples were also conducted to show reproducibility.
Randomization	N/A
Blinding	All bioinformatics analysis were blinded to samples and all samples in the same workflow went through same bioinformatics pipelines.

Reporting for specific materials, systems and methods

We require information from authors about some types of materials, experimental systems and methods used in many studies. Here, indicate whether each material, system or method listed is relevant to your study. If you are not sure if a list item applies to your research, read the appropriate section before selecting a response.

Materials & experimental systems

n/a	Involved in the study
☐	☒ Antibodies
☐	☒ Eukaryotic cell lines
☒	☐ Palaeontology and archaeology
☒	☐ Animals and other organisms
☒	☐ Clinical data
☒	☐ Dual use research of concern

Methods

n/a	Involved in the study
☒	☐ ChIP-seq
☐	☒ Flow cytometry
☒	☐ MRI-based neuroimaging

Antibodies

Antibodies used	All antibodies were purchased from Biolegend. CD3-FITC[SK7] (CAT# 344804), CD45-PerCP5.5[HI30] (CAT# 304028), CD19-BV421[HIB19] (CAT# 302233), CD14-PE-Cy7[HCD14] (CAT# 325618), CD66b-AF647[G10F5] (CAT# 305110), CD15-BV510[M-A251] (CAT# 356119), PI-PE (CAT#421301), EpCAM-PE[9C4] (CAT# 324206), CD16-APC[3G8] (CAT# 302011), CD11b-BV510[ICRF44] (CAT# 301334)
Validation	All antibodies have been validated by the manufacturer.

Eukaryotic cell lines

Policy information about cell lines and Sex and Gender in Research

Cell line source(s)	A549, NCI-H1975, NCI-H23 (H23), NCI-H522 (H522), NCI-H810, Hep G2 (HEPG2), SNU-182, SNU-449, SNU-387, Hep 3B2.1-7 (HEP3B2), BxPC-3, PANC-1, Kasumi-1, Reh and HTR-8/SVneo cell lines were purchased from ATCC. GM12878 cell line was obtained from the NIGMS Human Genetic Cell Repository at the Coriell Institute for Medical Research.
Authentication	GM12878 cells were authenticated through whole genome and whole exome sequencing. None of the other cell lines were authenticated.
Mycoplasma contamination	The cell lines were not tested for mycoplasma contamination.
Commonly misidentified lines (See ICLAC register)	None

Flow Cytometry

Plots

Confirm that:

☒ The axis labels state the marker and fluorochrome used (e.g. CD4-FITC).

☒ The axis scales are clearly visible. Include numbers along axes only for bottom left plot of group (a 'group' is an analysis of identical markers).

☒ All plots are contour plots with outliers or pseudocolor plots.

☒ A numerical value for number of cells or percentage (with statistics) is provided.

Methodology

Sample preparation	When applicable, cells were labeled with a panel of primary antibodies for 20 minutes at room temperature and washed twice. Propidium iodine was added to the cell mixture prior to acquisition and sorting.
Instrument	BD FACSMelody
Software	BD FACSChorus software was used to to collect the flow cytometry data. BD FlowJo was used to analyze the data.
Cell population abundance	1-90% depending on cell types in the sample.
Gating strategy	FSC and SSC were used to find viable, single cell events. Density plots with axes set on a log scale were used for gating. Distinct groups of positive and negative populations for individually stained populations were gated.

☐ Tick this box to confirm that a figure exemplifying the gating strategy is provided in the Supplementary Information.
